# Integrated transmission expansion planning incorporating fault current limiting devices and thyristor-controlled series compensation using meta-heuristic optimization techniques

**DOI:** 10.1038/s41598-024-63331-1

**Published:** 2024-06-06

**Authors:** Abdulaziz Almalaq, Khalid Alqunun, Rabeh Abbassi, Ziad M. Ali, Mohamed M. Refaat, Shady H. E. Abdel Aleem

**Affiliations:** 1 Department of Electrical Engineering, College of Engineering, University of Hail, 55473 Hail, Saudi Arabia; 2 Electrical Engineering Department, College of Engineering, Prince Sattam Bin Abdulaziz University, 11991 Wadi Addawaser, Saudi Arabia; 3 Electrical Engineering Department, Aswan Faculty of Engineering, Aswan University, Aswân, 81542 Egypt; 4 Photovoltaic Cells Department, Electronics Research Institute, Cairo, 11843 Egypt; 5 Department of Electrical Engineering, Institute of Aviation Engineering and Technology, Giza, 12658 Egypt

**Keywords:** Meta-heuristic algorithms, Transmission expansion planning, Fault current limiters, Thyristor-controlled series compensation devices, Electrical and electronic engineering, Energy infrastructure

## Abstract

Transmission expansion planning (TEP) is a vital process of ensuring power systems' reliable and efficient operation. The optimization of TEP is a complex challenge, necessitating the application of mathematical programming techniques and meta-heuristics. However, selecting the right optimization algorithm is crucial, as each algorithm has its strengths and limitations. Therefore, testing new optimization algorithms is essential to enhance the toolbox of methods. This paper presents a comprehensive study on the application of ten recent meta-heuristic algorithms for solving the TEP problem across three distinct power networks varying in scale. The ten meta-heuristic algorithms considered in this study include Sinh Cosh Optimizer, Walrus Optimizer, Snow Geese Algorithm, Triangulation Topology Aggregation Optimizer, Electric Eel Foraging Optimization, Kepler Optimization Algorithm (KOA), Dung Beetle Optimizer, Sea-Horse Optimizer, Special Relativity Search, and White Shark Optimizer (WSO). Three TEP models incorporating fault current limiters and thyristor-controlled series compensation devices are utilized to evaluate the performance of the meta-heuristic algorithms, each representing a different scale and complexity level. Factors such as convergence speed, solution quality, and scalability are considered in evaluating the algorithms’ performance. The results demonstrated that KOA achieved the best performance across all tested systems in terms of solution quality. KOA’s average value was 6.8% lower than the second-best algorithm in some case studies. Additionally, the results indicated that WSO required approximately 2–3 times less time than the other algorithms. However, despite WSO’s rapid convergence, its average solution value was comparatively higher than that of some other algorithms. In TEP, prioritizing solution quality is paramount over algorithm speed.

## Introduction

Transmission expansion planning (TEP) is the process of identifying and assessing the need for new transmission lines, substations, transformers, and associated facilities. The aim is to ensure that the transmission system can accommodate current and future electricity demand while maintaining stability and minimizing costs. This planning process takes various factors into account, including load growth projections, renewable energy integration, regulatory requirements, technological advancements, and economic considerations. TEP is a critical aspect of infrastructure development and investment decisions for electricity providers, policymakers, and system operators^1^.

TEP models can be classified into two types: deterministic and stochastic models^2,3^. Deterministic models provide insights into cost-effective solutions under deterministic conditions. They require precise data about future conditions and typically optimize the transmission system based on deterministic forecasts of load growth, generation capacity additions, and other relevant parameters. While stochastic models account for uncertainties and risk factors to enhance the reliability and resilience of plans. Stochastic models incorporate power network uncertainties such as load variations, renewable energy generation, and equipment failures. These models use stochastic optimization techniques such as stochastic programming, scenario-based optimization, and robust optimization to generate robust and resilient expansion plans.

The application of DC and AC optimal power flow-based TEP models is prevalent for conducting load flow analyses and evaluating the capabilities of generation units^1–3^. While DC models are commonly used, AC models are recognized for their superior accuracy and flexibility, enabling the incorporation of various technology models within the TEP framework. Abbasi et al.^4^ introduced an AC-based TEP approach (ACTEP) and compared its results with those of the DC model. Despite the higher costs associated with projects planned using ACTEP, the AC model is considered more technically suitable and closely aligned with actual system operations. Furthermore, the AC model facilitates the integration of reactive power planning and generation and transmission network expansion planning (into a unified problem, leading to cost reduction and enhanced system performance. Farrag et al.^5^ introduced two DCTEP models and one ACTEP model, illustrating that the AC model accurately represents power networks by appropriately considering factors such as generator capacity curves, node voltage limits, reactive power flow, and network losses during the planning phase. Abdi et al.^6^ proposed a mixed DC and AC planning model for TEP and RPP, employing DC power flow for TEP and AC power flow for RPP. Their findings highlight the superiority of the mixed model in reducing computational time and improving plan accuracy.

The TEP problem is a complex and challenging task that requires careful consideration of various factors to design an efficient and reliable transmission network^7,8^. For instance, TEP commonly has a multi-objective nature to balance conflicting objectives such as minimizing investment costs, reducing transmission losses, enhancing system reliability, and accommodating renewable energy integration. Additionally, the TEP problem involves a large number of decision variables such as the selection of new transmission lines, location of energy storage systems, transformer capacities, and network configurations, which contribute to the combinatorial nature of the problem. Furthermore, uncertainties related to future load patterns, generation availability, regulatory changes, and economic conditions further increase the complexity of TEP. These uncertainties require the use of probabilistic and scenario-based approaches in planning models to account for different potential scenarios and ensure robustness in the designed transmission network^9^. Additionally, the interdependency between transmission expansion and other aspects of power system planning, such as generation planning, grid operation, and market dynamics, adds another layer of complexity that necessitates coordinated and integrated planning approaches^10^. Addressing these complexities in TEP requires advanced optimization techniques, computational tools, data analytics, and stakeholder collaboration to develop optimal and resilient transmission network expansion plans that meet the evolving needs of modern power systems.

In solving the TEP model, various types of optimization algorithms are employed to efficiently search for optimal or near-optimal solutions within the complex and high-dimensional solution space^11^. These optimization algorithms can be broadly categorized into classical mathematical programming techniques and meta-heuristic algorithms. Classical mathematical programming techniques include linear programming, mixed-integer linear programming, quadratic programming, and nonlinear programming. These techniques are widely used in TEP to formulate and solve optimization problems with deterministic objectives and constraints, such as minimizing investment costs while meeting reliability criteria and operational constraints.

Meta-heuristic algorithms provide alternative approaches for addressing TEP problems, particularly when dealing with non-linear, non-convex, or large-scale optimization problems that involve uncertainties and complexities^12–16^. These algorithms are inspired by natural processes or social behavior and utilize heuristic search strategies to efficiently navigate solution spaces. They commonly employ population-based or swarm-based approaches to discover optimal solutions. Meta-heuristic algorithms are recognized for their adaptability, resilience, and ability to solve complex optimization problems with diverse objectives and constraints. The selection of an optimization algorithm is influenced by various factors, including the size and complexity of the problem, as well as the constraints and objectives involved^17–19^. Studies and benchmarking tests are often conducted to evaluate the performance of different meta-heuristic algorithms in solving TEP models across various scenarios and system conditions^20–22^. By utilizing a diverse set of optimization algorithms, TEP planners can explore a wide range of solution possibilities and make informed decisions to design cost-effective, reliable, and resilient transmission networks. Table 1 provides a summary of some meta-heuristic algorithms employed for solving TEP.

**Table 1:** Some metaheuristic algorithms applied to solve the TEP problem.

Optimization algorithm	Refs.	Year	Testing model	Testing system	Objective function
Ant colony optimization	Leeprechanon et al.^12^	2010	DC TEP	Garver network	Minimizing the cost of newly installed transmission lines
Harmony research	Verma et al.^13^	2010	DC TEP	IEEE 24-bus system South Brazilian 46 bus system	Minimizing the cost of newly installed transmission lines
Shuffled frog leaping algorithm	Eghbal et al.^14^	2011	DC TEP	IEEE 24-bus system	Minimizing the cost of newly installed transmission lines Minimizing congestion cost Minimizing load shedding values
Differential evolution algorithm	Alhamrouni et al.^15^	2014	AC TEP	Garver network IEEE 24-bus system	Minimizing the expansion cost of expanding the transmission network and adjusting generation capacity
Particle swarm optimization	Fathy et al.^16^	2017	DC TEP	Garver network The Egyptian West Delta network An Egyptian Extra High Voltage Network	Minimizing the cost of newly installed transmission lines
Non-dominated Sorting Genetic Algorithm II	Abbasi et al.^17^	2018	DC TEP	IEEE 24-bus system Iranian 400 kV transmission network	Minimizing investment costs Minimizing congestion costs Minimizing risk costs
Multi-Verse Optimizer	Shaheen et al.^18^	2019	DC TEP	The Egyptian West Delta network An Egyptian Extra High Voltage Network	Minimizing the expenses related to constructing new lines
Grey wolf optimization	Ghadimi et al.^19^	2021	AC TEP	Garver network	Minimizing the cost of newly installed lines Minimizing the cost of load disconnection penalties
• lévy flight distribution, • Sine cosine algorithm, • LSHADE-SPACMA	Refaat et al.^20^	2021	DC TEP	Egyptian West Delta network	Minimizing the expenses related to constructing new lines, fault current limiters, and generation units Minimizing generator operating costs Minimizing load shedding values
• Gravitational search algorithm • Imperialist competitive algorithm	Abdi et al.^6^	2022	DC TEP	IEEE 24-bus system IEEE 118- bus system	Minimizing the cost of newly installed lines Minimizing the cost of the installed reactive power sources
Hybrid snake optimization algorithm and sine cosine algorithm	Rawa et al.^21^	2022	ACTEP	Garver network IEEE 24-bus system	Minimizing the capital cost of newly installed lines, generation units, batteries and fault current limiters Minimizing the operating cost of generation units and batteries
Hybrid sine cosine artificial rabbits algorithm	Vellingiri et al.^22^	2023	ACTEP	Garver network IEEE 24-bus system	Improving the hosting capacity Minimizing the cost of newly installed lines and fault current limiters

**Figure 1:**
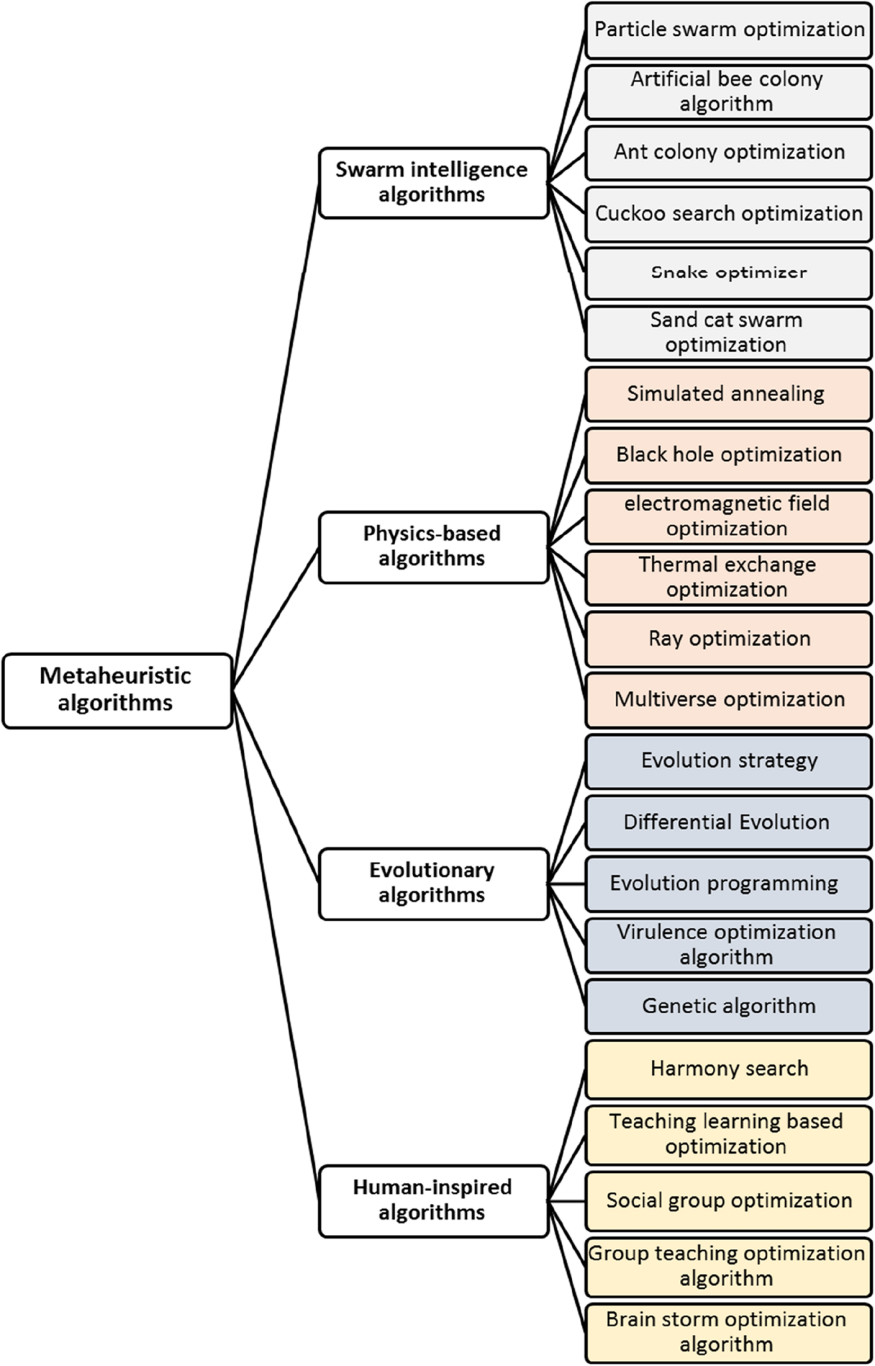
Classification of most common meta-heuristic optimization algorithms.

Figure 1 provides a summary of some of the most common meta-heuristic optimization algorithms. Recent advancements in meta-heuristic algorithms have been aimed at improving their efficiency, scalability, robustness, and adaptability to handle increasingly complex optimization tasks^23,24^. These algorithms have been integrated with other computational techniques like machine learning, deep learning, and optimization theory, giving rise to a new class of hybrid and adaptive algorithms. These algorithms capitalize on the unique strengths of each approach to solve complicated problems more efficiently than ever before. The ongoing research in meta-heuristic algorithms continues to explore innovative techniques, algorithms, and applications to further advance optimization science and engineering^25–27^.

As discussed previously, the optimization of TEP presents multifaceted challenges. However, selecting the most suitable optimization algorithm is crucial, considering that each algorithm has its own set of advantages and constraints. Therefore, exploring novel optimization algorithms is imperative to enrich the repertoire of available methodologies. The primary contributions of this paper are as follows:

- A comprehensive exploration of ten recent meta-heuristic algorithms for solving the TEP problem across three distinct power networks of varying scales.
- The ten meta-heuristic algorithms examined include Sinh Cosh Optimizer^28^, Walrus Optimizer^29^, Snow Geese Algorithm^30^, Triangulation Topology Aggregation Optimizer^31^, Electric Eel Foraging Optimization^32^, Kepler Optimization Algorithm^33^, Dung Beetle Optimizer^34^, Sea-Horse Optimizer^35^, Special Relativity Search^36^, and White Shark Optimizer^37^. These algorithms encompass a diverse array of search and optimization strategies, demonstrating potential across various optimization domains.
- Three distinct TEP models were proposed in this analysis to evaluate the performance of these algorithms. The first model adhered to the standard TEP model, concentrating on determining the optimal locations for new transmission lines and generation units. The second model expanded upon this by incorporating the planning model of thyristor-controlled series compensator (TCSC), thereby introducing additional decision-making variables. Finally, in the third model, the complexity was further heightened by integrating the planning models of TCSCs and fault current limiters (FCLs), resulting in a larger set of variables to be considered.

The subsequent sections of this paper are organized as follows: section “TEP models” presents the testing planning models used in this study. Section “Optimization algorithms” provides a summary of the operating mechanisms of the considered optimization algorithms. Section “Implementation of metaheuristics in solving TEP” delves into the strategy of implementing metaheuristics for solving TEP. In section “Testing systems”, the testing power networks are introduced. Section “Results and discussion” presents the results, while section “Conclusions” concludes the paper.

## TEP models

In this study, three TEP models are utilized to evaluate the performance of the optimization algorithms. These models differ in scale and the quantity of decision-making variables. The initial model illustrates the conventional TEP, which concentrates on identifying optimal locations for new transmission lines and generation facilities to accommodate projected load growth. In the second model, the integration of TEP with TCSC planning is examined. The third planning model integrates short-circuit current (SC) constraints and is designed to encompass planning considerations for transmission lines, generation units, TCSC installations, and FCL.

### Standard TEP model (model#1)

The conventional TEP methodology is formulated as a mixed-integer linear programming model, typically represented by (1)–(7)^16^. The primary goal of the objective function is to minimize the total investment cost associated with new transmission lines, alongside the operational and capital expenses related to generation units. This objective function can be expressed as follows:

1 $$Min.\;\;O.F_{1} = \mathop \sum \limits_{{\forall i,j \in\Omega _{B} }} C_{ij} \left( {N_{ij}^{l} - N_{ij}^{0} } \right) + \mathop \sum \limits_{{\forall G \in \Omega_{G} }} \left( {C_{inv,g} N_{G} P_{G}^{new} + C_{op,G} N_{G} P_{G} } \right)$$

While the problem constraints are explained by:

2 $$N_{ij}^{o} \le N_{ij}^{l} \le N_{ij}^{max} { };{ }\forall { }\left\{ {{ }i,j \in\Omega _{B} } \right\}$$

3 $$N_{G}^{min} \le N_{G} \le N_{G}^{max} { };{ }\forall { }\left\{ {{ }G \in\Omega _{G} } \right\}$$

4 $$P_{G}^{min} \le P_{G} \le P_{G}^{max} { };{ }\left\{ {{ }G \in\Omega _{G} } \right\}$$

5 $$N_{G} P_{G} - P_{d,i} = \mathop \sum \limits_{{\forall j \in\Omega _{B} }} P_{ij} { };{ }\forall { }\left\{ {j \in\Omega _{B} ;{ }G \in\Omega _{G} } \right\}$$

6 $$- { }N_{ij}^{l} P_{ij}^{max} \le \beta_{ij} { }N_{ij}^{l} { }\left( {\theta_{i} - \theta_{j} } \right) \le { }N_{ij}^{l} P_{ij}^{max} ;{ }\forall { }\left\{ {{ }i,j \in\Omega _{B} } \right\}$$

7 $$\theta_{i}^{min} \le \theta_{i} \le \theta_{i}^{max} ;{ }\forall { }\left\{ {{ }i \in\Omega _{B} } \right\}$$

where $N_{ij}^{l}$ and $N_{ij}^{0}$ denote the newly installed and existing circuits respectively, in a corridor between nodes *i* and *j*. $\Omega _{B}$ represents a set comprising all buses within the system. The boxed Eqs. (2)–(4) constrain the number of newly installed circuits and dictate the location and size of generation units within prescribed limits. $N_{G}$ signifies the count of generation units installed at the *G*th bus, while *P*_*G*_^*new*^denotes the capacity of the new generation unit in MW. $P_{G}$ represents the power dispatched from the generation units.

The power balance equation, expressed as Eq. (5), stipulates that the net power injected at any node must equate to the disparity between the power generated and consumed at that node. Equation (6) ensures that active power flowing through any line remains below its thermal limits $P_{ij}^{max}$, while Eq. (7) maintains the voltage angles $\theta_{i}$ of the buses within their designated thresholds. $\beta_{ij}$ symbolizes the susceptance of the transmission lines connecting bus *i* and bus *j*.

### TEP with TCSC planning model (model#2)

In the second model, the integration of TEP with TCSC planning is presented. It is formulated as a mixed-integer nonlinear programming model. The objective is to minimize the total investment cost associated with new transmission lines and TCSCs, while also considering the operational and capital expenses related to generation units. The objective function is given by:

8 $$Min.\;\;O.F_{2} = \mathop \sum \limits_{{\forall i,j \in {\Omega }_{B} }} C_{{ij}} \left( {N_{{ij}} ^{l} - N_{{ij}}^{0} } \right) + \mathop \sum \limits_{{\forall i,j \in {\Omega }_{{TCSC}} }} \left( {C_{1}^{{TCSC}} ~S_{{ij}}^{{TCSC2}} + C_{2}^{{TCSC}} ~S_{{ij}}^{{TCSC}} + C_{3}^{{TCSC}} } \right) + \mathop \sum \limits_{{\forall g \in \Omega _{G} }} \left( {C_{{inv,g}} ~N_{G} P_{G}^{{new}} + C_{{op,G}} N_{G} ~P_{G} } \right)$$

The problem constraints are detailed in (2)–(7) and further supplemented by (9) and (10). The second term in the objective function (8) accounts for the investment cost associated with installed TCSCs^38^. Introducing the TCSC module in series along any given route amplifies the equivalent capacity of power flow through that route by $\frac{{{ }N_{ij}^{l} \lambda_{ij}^{TCSC} }}{{1 - \lambda_{ij}^{TCSC} }}$. Here, $\lambda_{ij}^{TCSC}$ represents the requisite compensation level for a TCSC installed within the circuit between buses *i* and* j*.

9 $$\lambda_{min}^{TCSC} \le \lambda_{ij}^{TCSC} \le \lambda_{max}^{TCSC} { };{ }\forall { }\left\{ {i,j \in\Omega _{TCSC} } \right\}$$

10 $$- \left( {\frac{{{ }N_{ij}^{l} }}{{1 - \lambda_{ij}^{TCSC} }}} \right){ }P_{ij}^{max} \le \frac{{{ }N_{ij}^{l} { }\left( {\theta_{i} - \theta_{j} } \right)}}{{\left( {1 - \lambda_{ij}^{TCSC} } \right){ }X_{ij} }} \le \left( {\frac{{{ }N_{ij}^{l} }}{{1 - \lambda_{ij}^{TCSC} }}} \right){ }P_{ij}^{max} ;{ }\forall { }\left\{ {{ }i,j \in\Omega _{B} } \right\}$$

### TEP with TCSC and FCL planning model (model#3)

The model's objective is delineated through (11). It encompasses the investment and operational costs associated with various installed projects, whether they are FCLs, TCSCs, transmission lines, or newly installed generation units.

11 $$Min.\;\;O.F_{3} = \mathop \sum \limits_{{\forall i,j \in {\Omega }_{B} }} C_{{ij}} \left( {N_{{ij}} ^{l} - N_{{ij}}^{0} } \right) + \mathop \sum \limits_{{\forall i,j \in{\Omega }_{{TCSC}} }} \left( {C_{1}^{{TCSC}} ~S_{{ij}}^{{TCSC2}} + C_{2}^{{TCSC}} ~S_{{ij}}^{{TCSC}} + C_{3}^{{TCSC}} } \right) + \mathop \sum \limits_{{\forall g \in \Omega _{G} }} \left( {C_{{inv,g}} P_{g}^{{new}} + C_{{op,g}} ~P_{g} } \right) + \mathop \sum \limits_{{\forall i,j \in \Omega _{B} }} ~\left( {x_{{ij}}^{{FCL}} - ~x_{{ij}}^{{FCL - 1}} } \right)$$

The initial two terms aim to mitigate the investment expenses incurred in transmission and generation projects, as elucidated in (11). Meanwhile, the investment outlay for newly installed TCSCs is encapsulated in the third term. The fourth term accounts for the investment costs associated FCLs, necessary to maintain short-circuit current levels below the designated threshold value. $x_{ij}^{FCL}$ represents the calculated size of the FCL required between bus i and bus j to limit during abnormal operations.

The problem constraints are formulated in (2)–(7), along with Eqs. (9) and (10). Additionally, constraints (12) and (13) further restrict the problem. Short-circuit current constraints are delineated in (12) and (13). Various faults, including single line-to-ground faults, double line-to-ground faults, and line-to-line faults, frequently manifest in power networks. However, this study focuses on the worst-case scenario, the three-phase short-circuit fault. The short-circuit current level of substations is regulated by (13). Calculation of the short-circuit current is detailed in (14)^39–41^.

12 $$0 \le { }x_{ij}^{FCL} \le x_{ij,max}^{FCL} ;{ }\forall { }\left\{ {{ }s \in\Omega _{S} ,{ }i,j \in\Omega _{B} } \right\}$$

13 $$0 \le I_{i}^{SC} \le I_{max}^{SC} ;{ }\forall { }\left\{ {{ }s \in\Omega _{S} ,{ }i \in\Omega _{B} } \right\}$$

14 $$I_{i}^{SC} = \frac{{V_{i} \left( {\text{o}} \right)}}{{Z_{ii} }}$$

$V_{i}^{h} \left( o \right)$ is the pre-fault voltage, and $Z_{ii}$ is the bus *i* diagonal value in the impedance matrix. When an FCL module is installed in the route *m–n*, it is converted to a parallel impedance, which is obtained by (15)^40^.

15 $$z_{P} = \frac{{x_{mn} \left( {{ }x_{mn}^{FCL} + x_{mn} } \right)}}{{ - { }x_{mn}^{FCL} }}$$

## Optimization algorithms

### Sinh Cosh Optimizer (SCHO)

Bai et al.^28^ introduced the SCHO approach in 2023, leveraging the mathematical principles of Sinh and Cosh. SCHO comprises four key stages: two exploration phases, two exploitation phases, a bounded search strategy, and a switching mechanism. The operational framework of SCHO can be summarized as follows:

Similar to other metaheuristic algorithms, SCHO begins by randomly setting up a group of candidate solutions as provided in (16).

16 $$X = LB + \left( {UB - LB} \right).{ }rand$$

SCHO’s exploration stage is split into two phases during each iteration, and is necessary in the later iterations to avoid getting trapped in local optima. In the first phase, the new solution’s positions are updated by (17). While, in the second phase, the new solution’s positions are determined by (18). The threshold value (*T*) that triggers the transition between these phases is calculated using (19).

17 $$X^{t + 1} = \left\{ {\begin{array}{*{20}c} {X_{best} + r_{1} \times W_{1} \times X^{t} , if r_{2} > 0.5} \\ {X_{best} - r_{1} \times W_{1} \times X^{t} ,, if r_{2} < 0.5} \\ \end{array} } \right.$$

18 $$X^{t + 1} = \left\{ {\begin{array}{*{20}c} {X^{t} + \left| {\varepsilon \times W_{2} \times X_{best} - X^{t} } \right|, if r_{3} > 0.5} \\ {X^{t} - \left| {\varepsilon \times W_{2} \times X_{best} - X^{t} } \right|, if r_{3} < 0.5} \\ \end{array} } \right.$$

19 $$T = floor\left( {\frac{{iter^{Max} }}{ct}} \right)$$

where *iter*^*Max*^ represents the maximum value of iterations, *floor* is a MATLAB function that rounds down, and *ct* is a coefficient used to set the switching point between the two phases. The weight coefficient *W*_*1*_ determines the influence of *X*^*t*^ in the initial exploration stage, guiding potential solutions away from each other and towards the optimal solution as calculated by (20). Meanwhile, *W*_*2*_ represents the weight coefficient of *X*_*best*_ during the second exploration phase, determined by utilizing (21). *u* is a sensitive parameter that influences the precision of exploration during the initial phase. The values for a_1_ and a_2_ are determined as described in Bai et al.^28^. Whereas random numbers r_1_ to r_6_ fall within the range of 0 to 1.

20 $$W_{1} = r_{4} \times a_{1} \times \left( {\cosh r_{5} + u \sinh r_{5} - 1} \right)$$

21 $$W_{2} = r_{6} \times a_{2}$$

The exploitation process is divided into two distinct phases that occur consistently across all iterations. In the initial exploitation phase, exploitation is conducted in the proximity of *X*_*t*_, resulting in the formulation of the exploitation formula as depicted in Eq. (22). On the other hand, in the subsequent exploitation phase, candidate solutions delve further into exploiting the vicinity surrounding the currently best solution. The degree of exploitation around this optimal solution escalates with each iteration. The equation representing the position update function is presented in Eq. (23).

22 $$X^{t + 1} = \left\{ {\begin{array}{*{20}c} {X_{best} + r_{7} \times W_{3} \times X^{t} , if r_{8} > 0.5} \\ {X_{best} - r_{7} \times W_{3} \times X^{t} ,, if r_{8} < 0.5} \\ \end{array} } \right.$$

23 $$X^{t + 1} = X^{t} + r_{9} \times \frac{{\sinh r_{10} }}{{\cosh r_{10} }}\left| {W_{2} \times X_{best} - X^{t} } \right|$$

*W*_*3*_ is the weight coefficient responsible for guiding candidate solutions during the initial exploitation phase to explore the search space starting from nearby areas and extending towards farther regions. Its calculation is determined using (24). Random numbers *r*_*7*_ to *r*_*11*_ fall within the range of 0–1.

24 $$W_{3} = r_{11} \times a_{1} \times \left( {\cosh r_{11} + u \sinh r_{11} } \right)$$

To alternate between exploration and exploitation stages, a switching mechanism based on Sinh and Cosh functions is introduced. When A > 1, SCHO engages in exploration, whereas when A < 1, SCHO conducts exploitation. The values of A are determined according to the method described in Bai et al.^28^.

To maximize the exploration of the potential search space, the bounded search strategy is implemented. When SCHO employs the bounded search strategy consistently, the upper and lower bounds of optimization problems are determined using (25) for the upper bound and (26) for the lower bound. When the bounded search strategy is activated, all candidate solutions are randomly initialized within this potential space using Eq. (16). The initiation of this strategy is governed by *BS*_*k*_. The calculation of *BS*_*k*_ is detailed in Bai et al.^28^. X_second_ is the second optimal solution.

25 $$UB_{k} = X_{best} + \left( {1 - \frac{iter}{{iter^{Max} }}} \right) \times \left| {X_{best} - X_{second} } \right|$$

26 $$LB_{k} = X_{best} - \left( {1 - \frac{iter}{{iter^{Max} }}} \right) \times \left| {X_{best} - X_{second} } \right|$$

### Walrus Optimizer (WO)

WO was developed by Han et al.^29^ in 2023. It draws inspiration from the behaviours of walruses, which make decisions such as migration, breeding, roosting, feeding, gathering, and escaping based on receiving critical signals such as danger and safety signals. WO’s operating mechanism can be described as follows.

In the WO, the presence of a danger signal is utilized to determine whether the WO engages in exploration or exploitation. If the absolute value of the danger signal is equal to or greater than 1, the walrus herd relocates to a new area within the solution space, representing the exploration phase during the early stages of the algorithm. Conversely, during the later stages of the algorithm, the walrus herd engages in reproduction, indicating the exploitation phase. The security signal plays a crucial role in the exploitation phase as it influences the choice between roosting behaviour and foraging behaviour for individual walruses. Foraging behaviour encompasses two common actions, gathering and fleeing, both of which are regulated by the danger signals.

- Danger and safety signals

WO relies on danger and safety signals to determine the behaviour of walruses, which play a critical role in the decision-making process. The danger ($signal^{danger}$) and safety ($signal^{safety}$) signals, an essential component of WO, is defined as follows:

27 $$\left\{ {\begin{array}{*{20}c} {signal^{danger} = 2\left( {1 - \frac{t}{{iter^{max} }}} \right) \times \left( {2rand_{1} - 1} \right)} \\ {signal^{safety} = rand_{2} } \\ \end{array} } \right.$$

where $rand_{1}$ and $rand_{2}$ are randomly generated variables located in the range between 0 and 1.

- Migration (exploration)

In the migration phase, which signifies the exploration stage of the algorithm, the walrus's position is adjusted based on various parameters, including a random number *r*_*3*_, and two randomly selected solutions ($x_{m}^{t} ,x_{n}^{t}$). The equation used to update the walrus's position is as follows:

28 $$x_{i}^{t + 1} = x_{i}^{t} + \left( {x_{m}^{t} - x_{n}^{t} } \right) \times \left( {1 - \frac{1}{{1 - e^{{\frac{{ - 10\left( {t - {\raise0.7ex\hbox{${iter^{max} }$} \!\mathord{\left/ {\vphantom {{iter^{max} } 2}}\right.\kern-0pt} \!\lower0.7ex\hbox{$2$}}} \right)}}{{iter^{max} }}}} }}} \right) \times r_{3}^{2}$$

- Reproduction (exploitation)

When the risk factors are low, walrus herds tend to engage in breeding activities. During the reproduction phase, two main behaviours are observed: onshore roosting and underwater foraging. The mathematical model representing these behaviours is as follows:

1. Roosting behaviour.

The population of walruses consists of three categories: male, female, and juvenile individuals. These walruses update their positions using diverse methods:

- Dispersal of male walruses.

The Halton sequence distribution is employed for updating the position of male walruses. This distribution enables a more extensive distribution of the population within the search space.

(b) Dispersal of female walruses.

The behaviour of female walruses is influenced by two key factors: the male walruses ($male_{i}^{t}$) and the lead walrus ($x^{*}$). During the course of iterations, the influence of the female walrus's companion diminishes, while the influence of the leader becomes more prominent.

29 $$female_{i}^{t + 1} = female_{i}^{t} + \alpha \times \left( {male_{i}^{t} - female_{i}^{t} } \right) + \left( {1 - \alpha } \right) \times \left( {x^{*} - female_{i}^{t} } \right)$$

(c) Dispersal of juvenile walruses.

Young walruses often face the threat of predation from polar bears and killer whales near the edges of their colonies. Consequently, they must adapt their current positions in order to avoid being hunted.

30 $$young_{i}^{t + 1} = \left( {O_{i}^{t} - young_{i}^{t} } \right) \cdot P$$

where $young_{i}^{t + 1}$ denotes the updated position for the *i*th juvenile walrus. P signifies the distress coefficient of the juvenile walrus, which is a random number between 0 and 1. *O* stands for the reference safety position as provided in Han et al.^29^.

2. Foraging behaviour.

- Fleeing behaviour.

This behaviour arises during the later stages of the WO, and introducing a certain level of disturbance to the population aids walruses in engaging in worldwide exploration.

31 $$x_{i}^{t + 1} = x_{i}^{t} \times \left( {2rand_{1} - 1} \right) + \left( {x^{*} - x_{i}^{t} } \right) \times r_{4}^{2}$$

where r_4_ is a random number that falls within the interval of (0, 1).

(b) Gathering behaviour.

Walruses have the ability to collaborate in their search for food and navigation by taking cues from the movements of fellow walruses within the group. Sharing information about their whereabouts can greatly assist the entire herd in locating areas of the sea where food is more plentiful.

32 $$x_{i}^{t + 1} = \frac{{x_{1} + x_{2} }}{2}$$

where,

33 $$\left\{ {\begin{array}{*{20}c} {x_{1} = x^{*} - \left( {\beta r_{5} - \beta } \right) \times tan\left( \theta \right) \times \left| {x^{*} - x_{i}^{t} } \right|} \\ {x_{2} = x_{second}^{t} - \left( {\beta r_{5} - \beta } \right) \times tan\left( \theta \right) \times \left| {x_{second}^{t} - x_{i}^{t} } \right|} \\ \end{array} } \right.$$

*X*_*1*_ and *X*_*2*_ represent two factors influencing the foraging behaviour of walruses, while Xt denotes the position of the second walrus during the ongoing iteration. The variable r_5_ represents a random number within the interval (0, 1), and θ ranges from 0 to π.

### Snow Geese Algorithm (SGA)

The SGA was developed in 2023 by Tian et al.^30^. The algorithm takes inspiration from the migratory patterns of snow geese, replicating the unique “Herringbone” and “Straight Line” flight shapes observed during the geese’s migration. The symbol δ represents a hyper-parameter that applies a shift of the snow geese population from the exploration phase (which has a herringbone shape) to the exploitation phase (which is a straight line).

- A herringbone shape (exploration)

When δ is less than $\pi$, the SGA enters the exploration stage. In this stage, individuals within the populations are sorted based on their quality. Equation (34) is used to update the positions of individuals who exhibit exceptional fitness values and belong to the top 20%.

34 $$x^{t + 1} = x^{t} + \left( {4 \times rand - 2} \right) \times \left( {X^{*} - x^{t} } \right) + V^{t + 1}$$

where $V^{t + 1}$ is the next generation velocity and is calculated as follows:

35 $$V^{t + 1} = \frac{4 \times t}{{iter^{max} e^{{\frac{4 \times t}{{iter^{max} }}}} }} \times V^{t} + X^{*} - x^{t} - \frac{{1.29 \times \left( {V^{t} } \right)^{2} \times \sin {\delta } \times 10^{ - 2} }}{2}$$

Equation (36) is applied to update the positions of individuals who fall within the least fit quintile, including those who are weaker, unwell, or incapacitated and are located in the midsection of the population. The updating equation depends on the population central particle $x_{c}^{t}$ beside the optimal solution at the cuurent iteration $X^{*}$.

36 $$x^{t + 1} = x^{t} + \left( {4 \times rand - 2} \right) \times \left( {X^{*} - x^{t} } \right) - \left( {3 \times rand - 1.5} \right) \times \left( {x_{c}^{t} - x^{t} } \right) + V^{t + 1}$$

Finally, Eq. (37) is used to update the positions of individuals who remain in the population. The position represented by $x_{n}^{t}$ corresponds to the candidate solution. This candidate solution denotes the location of the lowest-ranked snow goose following population sorting.

37 $$x^{t + 1} = x^{t} + \left( {4 \times rand - 2} \right) \times \left( {X^{*} - x^{t} } \right) + \left( {3 \times rand - 1.5} \right) \times \left( {x_{c}^{t} - x^{t} } \right) - \left( {2 \times rand - 1} \right) \times \left( {x_{n}^{t} + x^{t} } \right) + V^{t + 1}$$

- A straight-line shape (exploitation)

During this stage, the algorithm places greater emphasis on avoiding local optima rather than exact navigation. Two strategies are employed by snow geese as they adopt a straight-line flight pattern. The individuals’ new position is determined as follows:

38 $$x^{t + 1} = \left\{ {\begin{array}{*{20}c} {x^{t} + r \times \left( {x^{t} - X^{*} } \right), if r > 0.5 } \\ {X^{*} + \left( {x^{t} - X^{*} } \right) \times r \oplus Brownian\left( d \right) if r \le 0.5 } \\ \end{array} } \right.$$

where *r* is a random number, and $\oplus$ indicates to entry wise multiplication.

### Triangulation Topology Aggregation Optimizer (TTAO)

The TTAO was developed in 2023 by Zhao et al.^31^. The TTAO algorithm uses similar triangles in its approach. Through iterative evolution, new vertices are constantly generated to form similar triangles of varying sizes. Each triangle in the TTAO algorithm is seen as a basic evolutionary unit, consisting of four agents—three vertices of the triangle and one random vertex inside. Additionally, the algorithm utilizes aggregation to group vertices with superior characteristics. The TTAO algorithm uses aggregation to collect vertexes with good information within or between different topological units. Note that all constructed triangles in the algorithm are equilateral and derived from the second theorem for constructing similar triangles.

The TTAO algorithm comprises of two techniques, namely the general aggregation and the local aggregation. Both techniques work together to create multiple triangular topological units that are similar to each other, through iterative processes. This helps to balance the exploration and exploitation in the algorithm.

- Generic aggregation

During the exploration phase of generic aggregation, the focus is put on gathering information of good individuals in various triangular units, which is then combined to create new feasible solutions. The process involves an exchange of information between the best individual in each triangular topological unit and the best individual in any randomly selected set of units. The better two-vertex connection produces the newly individual, which can be expressed mathematically as follows:

39 $$x_{i, new1}^{t + 1} = r \times x_{i}^{t,*} + \left( {1 - r} \right) \times x_{random}^{t,*}$$

where r is a randomly generated number within the range of 0 to 1. $x_{i}^{t,*}$ represents the best position for unit i, while $x_{random}^{t,*}$ represents a randomly chosen unit at that iteration.

- Local aggregation

Local aggregation primarily focuses on the exploitation stage. During this phase, triangular topological components are grouped together internally. Following the earlier phase, a triangular structure was created temporarily among the improved optimal or suboptimal individuals and the two vertices within the group exhibiting high fitness levels. The new vertex is determined as follows:

40 $$x_{i, new2}^{t + 1} = x_{i}^{t + 1,*} + \ln \left( {\frac{{e - e^{3} }}{{iter^{max} - 1}}t + e^{3} - \frac{{e - e^{3} }}{{iter^{max} - 1}}} \right) \times x_{s}^{t + 1,*}$$

where $x_{s}^{t + 1,*}$ represents the individual with the best suboptimal performance at the *i* ^th^ iteration. It is equal to $x_{i, new1}^{t + 1}$ if the fitness value of $x_{i, new1}^{t + 1}$ is better than the fitness value of $x_{s}^{t,*}$. Otherwise, it equals $x_{s}^{t,*}$.

### Electric Eel Foraging Optimization (EEFO)

The EEFO was developed in 2023 by Zhao et al.^32^. It takes inspiration from the collective foraging behaviours of electric eels, aiming to mimic four essential foraging behaviours—interaction, resting, hunting, and migration—in its mathematical model. This approach aims to facilitate both exploration and exploitation in the optimization process.

The EEFO algorithm employs an energy factor to govern the search behaviours, facilitating a balanced transition between exploration (Interacting behaviour) and exploitation (resting, hunting, and migration behaviours) for enhanced optimization performance. The energy factor of an eel plays a crucial role in selecting the appropriate strategy, whether it is exploration or exploitation. The energy factor is precisely defined as follows:

41 $$E^{t} = 4 \times {\text{sin}}\left( {1 - \frac{t}{{iter^{max} }}} \right) \times {\text{ln}}\left( \frac{1}{r} \right)$$

where *r* is a random number between 0 and 1. When E is greater than1, the exploration stage is applied. Otherwise, the exploration phase is employed.

- Interacting behaviour

When eels come across a group of fish, they engage in swimming and stirring movements together. Subsequently, they form a large electrified loop in the water to ensnare multiple small fish at the centre of the loop. This activity can be seen as the exploration phase. The updating equation for individuals in this stage can be expressed as follows:

42 $$\left\{\begin{array}{c}if F({x}_{j}^{t})<F({x}_{i}^{t})\to \left\{\begin{array}{c}{x}_{i}^{t+1}={x}_{j}^{t}+C\times \left({\overline{x} }^{t}-{x}_{i}^{t}\right), if {p}_{1}>0.5\\ {x}_{i}^{t+1}={x}_{j}^{t}+C\times \left({x}_{r}^{t}-{x}_{i}^{t}\right), if {p}_{1}\le 0.5\end{array}\right.\\ if F({x}_{j}^{t})\ge F({x}_{i}^{t})\to \left\{\begin{array}{c}{x}_{i}^{t+1}={x}_{i}^{t}+C\times \left({\overline{x} }^{t}-{x}_{j}^{t}\right), if {p}_{2}>0.5\\ {x}_{i}^{t+1}={x}_{i}^{t}+C\times \left({x}_{r}^{t}-{x}_{j}^{t}\right), if {p}_{2}\le 0.5\end{array}\right.\end{array}\right.$$

where $\overline{x}^{t} = \frac{1}{np}\mathop \sum \limits_{i = 1}^{np} x_{i}^{t}$, and $x_{r}^{t} = LB + r \times \left( {UP - LB} \right)$. $p_{1}$ and $p_{2}$ represent random numbers between 0 and 1, $F\left( {x_{i}^{t} } \right)$ denotes the fitness of the candidate position of the *i*th electric eel, $x_{j}^{t}$ is the position of an eel chosen randomly from the current population, and r is a random vector ranging between 0 and 1. C represents the random movement of eels, and it is calculated as explained in Zhao et al.^32^.

- Resting behaviour

In order for electric eels to exhibit resting behaviour in EEFO, the resting area needs to be set up beforehand. To improve the search efficiency, a designated resting area is set up in the area where a single dimension of the eel's position vector aligns with the main diagonal within the search space. Once the resting area is identified, the eels will relocate to it for resting. An eel moves towards its resting spot by adjusting its position relative to its designated resting area. The behaviour of resting can be described as:

43 $$x_{i}^{t + 1} = Y_{i}^{t + 1} + n_{2} \times \left( {Y_{i}^{t + 1} \times round\left( {rand} \right) \times x_{i}^{t} } \right)$$

where,

44 $$Y_{i}^{t + 1} = Z^{t} + \alpha \times \left| {Z^{t} - x^{*} } \right|$$

More details about $Z^{t}$ and $\alpha$ is presented in Zhao et al.^32^.

- Hunting behaviour

Once the hunting area is established, an electric eel initiates its hunting activities within that specific region. The hunting behaviour observed in EEFO algorithm includes a curling movement. This curling behaviour demonstrated by the eels during hunting can be summarized as follows:

45 $$x_{i}^{t + 1} = H_{prey}^{t + 1} + \eta \times \left( {H_{prey}^{t + 1} - round\left( {rand} \right) \times x_{i}^{t} } \right)$$

where η denotes the curling factor. The calculation of η and $H_{prey}^{t + 1}$ is presented in Zhao et al.^32^.

- Migrating

The migration behaviour of eels from the resting area to the hunting area, when they detect prey, is expressed through:

46 $$x_{i}^{t + 1} = - r_{1} \times Y_{i}^{t + 1} + r_{2} \times H_{r}^{t + 1} - L \times \left( {H_{r}^{t + 1} - x_{i}^{t} } \right)$$

$r_{1}$ and $r_{2}$ are random values in the range between 0 and 1. More details about $H_{r}^{t + 1}$ and L can be found in Zhao et al.^32^.

### Kepler optimization algorithm (KOA)

The KOA algorithm was developed by Abdel-Basset et al.^33^ in 2023. Kepler's three laws of planetary motion describe key aspects of how planets move around the sun, focusing on elliptical orbits, equal areas swept out in equal time intervals, and the relationship between orbital period and semi-major axis. Inspired by these laws, Abdel Basset et al. developed the KOA metaheuristic algorithm, which represents planets and the sun as solutions to optimization problems. KOA utilizes the dynamic positional interactions between planets and the sun over time, guided by Kepler's principles.

The updating mechanism of the KOA involves two distinct stages, outlined as follows. In the initial stage, KOA computes the planet's updated position utilizing (47). The adjustment in the planet's velocity direction, indicated by ∂, incorporates a random scalar, *r*, drawn from a standard normal distribution. Here, $x_{s}^{t}$ denotes the current position of the sun, serving as the benchmark for the optimal solution. Meanwhile, $v_{i}^{t}$ signifies the velocity of the planet at time t, and $f_{i}^{g}$ represents the gravitational force. The computation formulas for $v_{i}^{t}$, $f_{i}^{g}$, $\partial$, and $\cup$ are detailed in Abdel-Basset et al.^33^.

47 $$x_{i}^{t + 1} = x_{i}^{t} + \partial \times v_{i}^{t} + \left( {f_{i}^{g} + \left| r \right|} \right) \times \cup \times \left( {x_{s}^{t} - x_{i}^{t} } \right)$$

In the second phase of the KOA, the adjustment of planet positions near the sun—regarded as the optimal solution—is executed using Eq. (48). Within this stage, the adaptive factor denoted as *h*, as defined in Abdel-Basset et al.^33^, assumes a crucial role. The value of *h* changes gradually over time. When *h* is high, the exploration operator is used to increase the distance between the planets and the Sun. Conversely, when *h* is low, the exploitation operator is utilized to optimize areas near the current best solution if the distance between the Sun and the planets is short. The variables* r* and *r4* respectively embody a random number adhering to a normal distribution and a random value spanning from 0 to 1. Additionally, $x_{a}^{t}$ and $x_{b}^{t}$ represent two randomly generated solutions.

48 $$x_{i}^{t + 1} = x_{i}^{t} \times \cup_{1} + \left( {1 - \cup_{1} } \right) \times \left( {\left( {\frac{{x_{i}^{t} + x_{s}^{t} + x_{a}^{t} }}{3}} \right) + h \times \left( {\frac{{x_{i}^{t} + x_{s}^{t} + x_{a}^{t} }}{3} - x_{b}^{t} } \right)} \right)$$

### Dung Beetle Optimizer (DBO)

The DBO algorithm was developed by Xue et al.^34^ in 2022. It is an innovative population intelligence algorithm that takes inspiration from the diverse behaviours of dung beetles. The algorithm is renowned for its robust capability in seeking merit and achieving rapid convergence. It comprises four primary processes: ball rolling, breeding, foraging, and stealing.

- Ball rolling process

In scenarios where dung beetles encounter unhindered ball rolling, it is hypothesized that the intensity of light impacts the beetles' positioning. As a result, the formula for updating the dung beetle's position is expressed as follows:

49 $$X^{t + 1} = X^{t} \times + \alpha k X^{t - 1} + b \left| {X^{t} - X^{w} } \right|$$

The deflection coefficient’s constant value is represented by k, which falls within the range of (0, 0.2]. The constant value b is assigned a value of (0, 1), while α is assigned the natural coefficient of − 1 or 1. *X*^*w*^ represents the worst position of the ball.

When encountering an obstacle, the dung beetle adapts by performing a dance to locate an alternative route. The dancing behaviour is modelled using a tangent function in the algorithm. The angle tilted from the direction of [0, π] is represented by the symbol $\emptyset$. After identifying a new direction and rolling the ball, the dung beetle's location is updated as follows:

50 $$X^{t + 1} = X^{t} + \tan \emptyset \left| {X^{t} - X^{t - 1} } \right|$$

- Breeding process

Female dung beetles roll their dung balls to a secure location while concealing them in order to make them more suitable for laying their eggs in a favourable habitat. The limits of the area where the brood balls are placed can be described as follows:

51 $$\left\{ {\begin{array}{*{20}c} {LB^{*} = max\left( {X^{*} \times \left( {1 - R} \right), LB} \right)} \\ {UB^{*} = max\left( {X^{*} \times \left( {1 - R} \right), UB} \right)} \\ \end{array} } \right.$$

where $X^{*}$ represents the current optimal solution, while LB^*^ and UB^*^ represent the spawning area’s lower and upper boundaries. *R* = *1−t/iter*^*max*^. The spawning area is determined by the female dung beetle, and only one egg is laid at a time. The breeding behavior equation has been updated and can be expressed as follows:

52 $$B^{t + 1} = X^{*} + b_{1} \times \left( {B^{t} - LB^{*} } \right) + b_{2} \times \left( {B^{t} - UB^{*} } \right)$$

The position of the brood ball at each iteration is denoted by B^t+1^, where b_1_ and b_2_ are composed of random independent vectors. However, it is crucial to confine the position of brood balls within the spawning area.

- Foraging process

The adult dung beetles emerge from the ground to search for food after their growth from small beetles. Additionally, the foraging area is constantly updated with the number of iterations using the following equation:

53 $$\left\{ {\begin{array}{*{20}c} {LB^{b} = max\left( {X^{g*} \times \left( {1 - R} \right), LB} \right)} \\ {UB^{b} = max\left( {X^{g*} \times \left( {1 - R} \right), UB} \right)} \\ \end{array} } \right.$$

The term $X^{g*}$ represents the position of the best global solution, while the optimal foraging area's lower and upper bounds are denoted by $LB^{b}$ and $UB^{b}$, respectively. The location updating equation can be written as follows:

54 $$x^{t + 1} = x^{t} + C_{1} \times \left( {x^{t} - LB^{b} } \right) + b_{2} \times \left( {B^{t} - UB^{b} } \right)$$

where *C*1 represents a random number that follows a normal distribution, while *C*2 is a random vector that is defined on the interval (0, 1).

- Stealing process

There are certain dung beetles that have been labelled as thieves within their population. These beetles steal dung balls from other beetles. It is possible for the position of these thieving beetles to change as follows:

55 $$x^{t + 1} = X^{g*} + \rho \times \sigma \times \left( {\left| {x^{t} - X^{*} } \right| + \left| {x^{t} - X^{g*} } \right|} \right)$$

The symbol $\sigma$ represents a vector of random values that follows a normal distribution. The letter $\rho$ represents a fixed value.

### Sea-Horse Optimizer (SHO)

The SHO was developed in 2022 by Zhao et al.^35^. SHO draws inspiration from the natural behaviours of seahorses, particularly their movement patterns, predation strategies, and breeding habits. These three intelligent behaviours are translated into mathematical expressions to ensure a balance between local exploitation and global exploration within the SHO algorithm.

- The movement behavior

The various movement patterns exhibited by sea horses roughly adhere to the normal distribution *randn* (0, 1). To balance the exploration and exploitation aspects, r1 is set to 0 as the threshold point, allocating half for local exploration and the remaining half for global search. The movements can be categorized into two cases. When the normal random value r1 falls on the right side of the cut-off point, the first case is employed. Conversely, when the random value r1 falls on the left side of the cut-off point, the second case is executed. The generation of a sea horse's new position can be mathematically formulated as follows:

56 $$x_{new1}^{t + 1} = \left\{ {\begin{array}{*{20}c} {x^{t} + levy\left( z \right) \times \left( {\left( {x^{*} - X^{t} } \right) \times m \times y \times z + x^{*} } \right), if r_{1} > 0 } \\ {x^{t} + rand \times l \times \beta_{t} \left( {x^{*} - \beta_{t} \times X^{t} } \right) if r_{1} \le 0 } \\ \end{array} } \right.$$

where $= 0.05e^{0.05\vartheta } \times {\text{cos}}\left( \vartheta \right)$, $y = 0.05e^{0.05\vartheta } \times {\text{sin}}\left( \vartheta \right)$, and $z = 0.05e^{0.05\vartheta } \times \vartheta$. $\vartheta$ is a random value that takes a value between 0 and 2π. $levy\left( z \right)$ is Lévy flight distribution function. $l$ represents the constant coefficient, while $\beta_{t}$ denotes the random walk coefficient associated with Brownian motion.

- The predation behavior

The sea horse has two potential outcomes when preying on zooplankton and small crustaceans: success and failure. The random number r2 within SHO is configured to delineate these outcomes, set to a critical value of 0.1. If r2 > 0.1, it signifies a successful predation; otherwise, it signifies a failed predation. The mathematical expression encapsulating this predation behaviour is as follows:

57 $$x_{new2}^{t + 1} = \left\{ {\begin{array}{*{20}c} {\alpha \times \left( {x^{*} - rand \times x_{new1}^{t + 1} } \right) + \left( {1 - \alpha } \right) \times x^{*} , if r_{2} > 0.1 } \\ {\left( {1 - \alpha } \right) \times \left( {x_{new1}^{t + 1} - rand \times x^{*} } \right) + \alpha \times x_{new1}^{t + 1} , if r_{2} \le 0.1 } \\ \end{array} } \right.$$

where $$\alpha = \left( {1 - \frac{t}{{iter^{max} }}} \right)^{{\frac{2t}{{iter^{max} }}}}$$.

- The breeding behavior

The population is divided into male and female groups based on their fitness levels. It's important to note that, given the breeding responsibility of male sea horses, the SHO algorithm selects half of the individuals with the highest fitness values as fathers and the remaining half as mothers. Male and female sea horses are paired randomly to generate offspring. To streamline the implementation of the proposed SHO algorithm, it is assumed that each pair of sea horses produces only one offspring. The expression for the offspring is as follows:

58 $$x^{offspring} = r_{3} \times x^{father} + \left( {1 - r_{3} } \right) \times x^{mother}$$

where *r3* is a random number within the range [0, 1]. $x^{father}$ and $x^{mother}$ denote randomly chosen individuals from the male and female populations, respectively.

### Special relativity search (SRS)

The SRS was developed in 2022 by Goodarzimehr et al.^36^. It draws its inspiration from the interactions observed among particles within an electromagnetic field. These interactions are assessed through the application of the Lorentz force, and the equation of motion is formulated utilizing angular frequency. The magnetic force acting between particles operates perpendicular to both the velocity of charged particles and the magnetic field, resulting in a circular trajectory for the particles. Uniquely, this approach incorporates principles from the theory of special relativity physics to calculate the coordinates of charged particles within each rotation for the first time. The primary equation of the SRS is derived by incorporating two key phenomena: length contraction and time dilation.

Mathematically, the SRS can be formulated as follows. The particle-to-particle distance ($D_{ij}^{t}$) in the magnetic field is calculated by employing the Euclidean norm as defined in (59).

59 $$D_{ij}^{t} = norm\left( {x_{i}^{t} - x_{j}^{t} } \right)$$

Then, the charge of each particle ($Q_{i}^{t}$) can be expressed as:

60 $$Q_{i,j}^{t} = \frac{{F_{i,j}^{t} - F_{worst}^{t} }}{{F_{gbest}^{t} - F_{worst}^{t} }}$$

where $F_{i,j}^{t}$ represents the fitness value of particle $x_{i}^{t}$ or the particle $x_{j}^{t}$. $F_{gbest}^{t}$ and $F_{worst}^{t}$ denote the global best and worst solutions in the population, respectively.

The frequency of the cyclotron is determined by employing (61). Where *m* is the particle's mass.

61 $$\omega_{n} = {\upmu }\frac{{Q_{i}^{t} Q_{j}^{t} }}{{m D_{ij}^{3} }} v_{j}$$

The particles' new coordinates can be obtained by:

62 $$x_{j} = x_{j} + \frac{v}{{\omega_{n} }} \times \sin \left( {\omega_{n} } \right)\to ^{{v = \omega_{n} D_{ij} }} \frac{{\omega_{n} D_{ij} }}{{\omega_{n} }} \times \sin \left( {\omega_{n} } \right) = D_{ij} \sin \left( {\omega_{n} } \right)$$

The new solutions of the population can determined by (63). In this algorithm, $\beta$ is less than one and is set equal to a random number between 0 and 1.

63 $$x^{t + 1} = \beta^{2} \times x^{t} + \left( {{\upmu }\frac{{Q_{i}^{t} Q_{j}^{t} }}{{m D_{ij}^{2} }} v_{j} } \right) \times \sqrt {1 - \beta^{2} } + x_{j}^{t} \times \sqrt {1 - \beta^{2} }$$

### White Shark Optimizer (WSO)

The WSO was developed in 2022 by Braik et al.^37^. The fundamental concepts and foundations of WSO draw inspiration from the behaviours exhibited by great white sharks. Specifically, their remarkable abilities in hearing and smelling during navigation and foraging serve as the basis for mathematical modelling. These behavioural aspects are incorporated to ensure a suitable equilibrium between exploration and exploitation within WSO. This enables the search agents to effectively explore and exploit various regions of the search space, ultimately facilitating optimization.

Identifying the optimal solutions is achieved through the following behaviours:

- Movement speed towards prey

A white shark identifies the location of its prey by detecting a pause in the waves caused by the prey's movement, as depicted in (64).

64 $$v_{i}^{t + 1} = \in \left( {v_{i}^{t} + P_{1} \left( {x_{gbest}^{t} - x_{i}^{t} } \right) \times c_{1} + P_{2} \left( {x_{gbest}^{{v_{i}^{t} }} - \omega_{i}^{t} } \right) \times c_{2} } \right)$$

where $= \frac{2}{{\left| {2 - \tau - \sqrt {\tau^{2} - 4\tau } } \right|}}$. *τ* indicates to the accelerating factor that is set to 4.125. $v_{i}^{t}$ represents the velocity vector of the ith white shark in the *t* iteration. $x_{gbest}^{{v_{i}^{t} }}$ represents the best-known position vector for the *i*th white shark within the swarm. Additionally, *c*_*1*_ and *c*_*2*_ are two randomly generated values uniformly distributed in the range [0, 1]. $P_{1}$ and $P_{2}$ are calculated using (65). The values for $P^{min}$ and $P^{max}$ are determined as 0.5 and 1.5, respectively.

65 $$\left\{ {\begin{array}{*{20}c} {P_{1} = P^{max} + \left( {P^{max} - P^{min} } \right) \times e^{{ - \left( {4t/iter^{max} } \right)^{2} }} } \\ {P_{2} = P^{min} + \left( {P^{max} - P^{min} } \right) \times e^{{ - \left( {4t/iter^{max} } \right)^{2} }} } \\ \end{array} } \right.$$

- Movement towards optimal prey

In this particular context, the behaviour of white sharks approaching their prey was described using the position-updating strategy outlined as follows:

66 $$x_{i}^{t + 1} = \left\{ {\begin{array}{*{20}c} {x_{i}^{t} . \to \oplus x_{o} + u.a + l.b ;if rand < mv} \\ {x_{i}^{t} + {\raise0.7ex\hbox{${v_{i}^{t} }$} \!\mathord{\left/ {\vphantom {{v_{i}^{t} } f}}\right.\kern-0pt} \!\lower0.7ex\hbox{$f$}} ;if rand \ge mv} \\ \end{array} } \right.$$

The symbol ⊕  represents a bitwise XOR operation. The frequency of the white shark’s wavy motion is denoted by *f*, and rand represents a randomly generated number uniformly distributed in the range [0, 1]. The parameter $mv$ is introduced to quantify the intensity of the white shark’s sensory perception, specifically its hearing and olfactory abilities, which gradually increase with each iteration. More details can be found in Braik et al.^37^.

- Movement towards the best white shark

Great white sharks possess the ability to sustain their position towards the nearest best solution in proximity to the prey. This behaviour is mathematically formulated as:

67 $${}^{\prime }x_{i}^{{t + 1}} = ~x_{{gbest}}^{t} + r_{1} \times \left| {rand \times \left( {x_{{gbest}}^{t} - x_{i}^{t} } \right)} \right| \times sgn\left( {r_{2} - 0.5} \right);~~if~rand < s_{s}$$

where ${}^{\prime }x_{i}^{{t + 1}}$ represents the revised location of the *i*th white shark relative to the prey's position. *r1, r2*, and rand are random values within the interval [0, 1]. $s_{s}$ is a parameter proposed to indicate the effectiveness of smell and sight senses in white sharks as they trail other white sharks near ideal prey.

- Fish school behaviour

The behaviour of fish schools of white sharks is characterized by the following formula:

68 $$x_{i}^{{t + 1}} = ~\frac{{x_{i}^{t} + {}^{\prime }x_{i}^{{t + 1}} }}{{2 \times rand}}$$

The sharks can adapt their positions according to the leading shark that reaches the vicinity of the target, optimizing their location. The final destination of the sharks ideally surrounds the prey within the search area. The collective behaviour of WSO is characterized by fish movements and the sharks' alignment with the superior shark, enhancing both local and global search abilities.

## Implementation of metaheuristics in solving TEP

**Figure 2:**
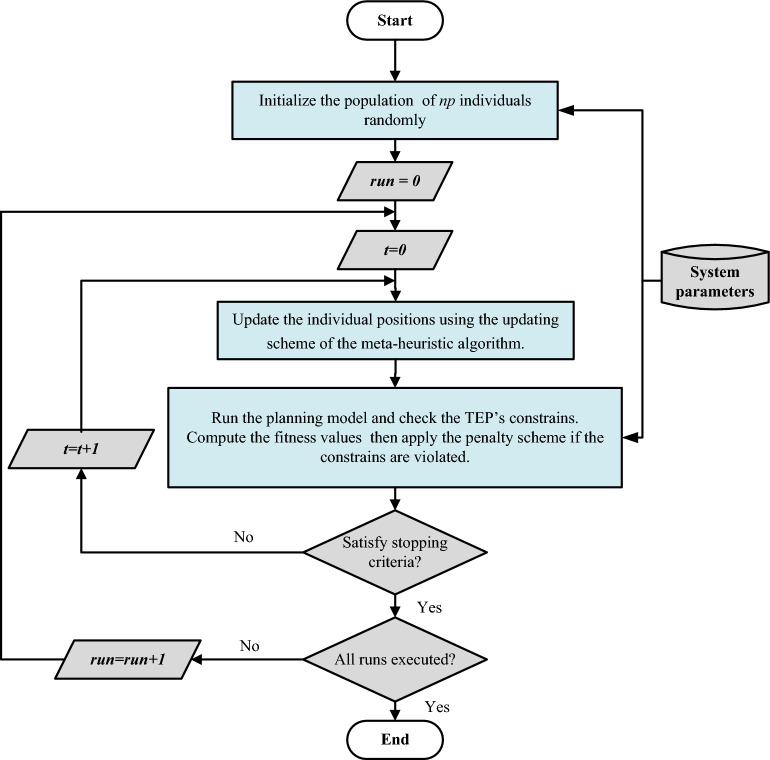
Steps of the application of meta-heuristics in solving the TEP problem.

The mechanism of operation of meta-heuristics in solving the TEP problem is described in Fig. 2. It comprises several pivotal stages. Initially, data concerning generation and transmission lines are collated, and their boundaries are defined. Subsequently, an initial population is randomly generated, ensuring adherence to these boundaries. Throughout each iteration, the positions of individuals are adjusted according to the algorithm's updating scheme, while concurrently, the objective function is assessed to ascertain the optimal solution. Any deviations from operating constraints result in significant penalization. These procedures persist until a predefined stopping criterion, often the maximum iteration limit, is met. This iterative cycle is then reiterated until the specified number of runs is accomplished, ultimately culminating in the identification of the optimal network configuration yielded by the best run. The operating mechanism of TEP-based metaheuristics for solving strategies can be summarized as follows:

**Step 1:** The data for the generation and transmission lines of the network are first prepared, and their lower and upper bounds are set.

**Step 2:** The initial population is randomly generated considering the lower and upper bounds of the decision-making variables as provided in (16).

**Step 3:** In each iteration, the following steps are carried out:

- The position of each individual in the population is updated using the updating scheme of the meta-heuristic algorithm.
- The objective function is calculated, and the best solution is defined. If the candidate solutions do not meet the operating constraints, a high penal value is added to the objective function.
- Repeat a and b until the stopping criterion is achieved (i.e., the maximum number of iterations is conducted).

**Step 4:** Repeat steps (1–3) until the maximum number of runs are conducted.

**Step 5:** Determine the best run that gives the best configuration of the network.

## Testing systems

**Figure 3:**
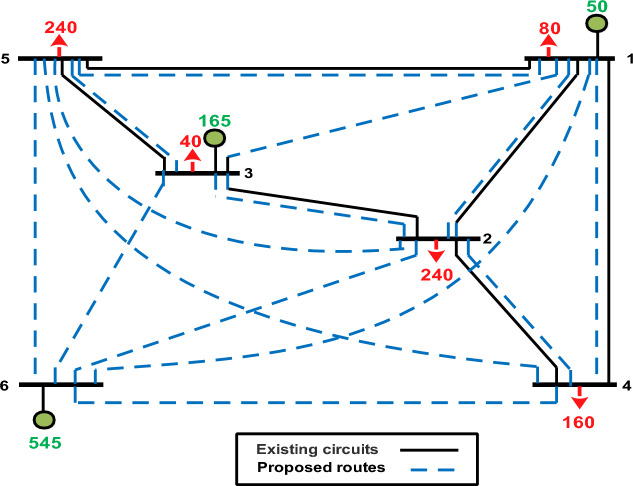
Single-line diagram of the Garver system.

The optimization algorithms under consideration are tested using both the Garver Network, the Egyptian West Delta Network (WDN), and the IEEE 118-bus system. The initial configuration of the Garver Network is illustrated in Fig. 3, comprising 15 power routes and 6 nodes, with a total power demand of 760 MW. System data can be found in^42^. In Fig. 3, candidate routes are depicted by dotted lines, while existing routes are represented by solid lines.

**Figure 4:**
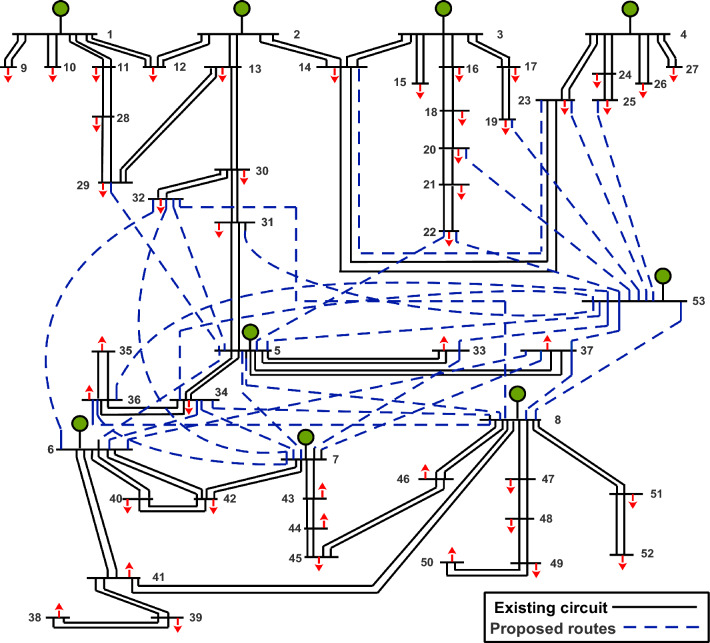
Single-line diagram of WDN.

The WDN serves as an Egyptian sub-transmission network, with its initial configuration shown in Fig. 4 and system data provided in^16^. It encompasses 52 buses and 55 routes, each equipped with two circuits. Plans include the installation of a new generation station at bus number 53 to accommodate anticipated load growth^16^. In Fig. 4, candidate routes are indicated by dotted lines, while existing routes are delineated by solid lines.

The 118-bus system encompasses 118 nodes, 54 thermal generation stations, and 186 pre-existing lines^43^. With a total load reaching 6.886 GW, the proposal entails installing a new circuit along each route.

## Results and discussion

The simulations were executed on the MATLAB r2021a platform using a DELL PC model named OptiPlex7050, equipped with an Intel® Core™ i7 CPU running at 2.6 GHz and 16 GB RAM. In total, 20 simulation runs were executed to ensure a thorough analysis, thereby enhancing the statistical reliability of the results. The maximum number of iterations was set to 300. The capital and operation cost coefficients of generation units are given in^44^, while the cost coefficient parameters of the TCSC are provided in^38^. The cost coefficient of the FCL module is introduced in^45^.

### The Garver network

#### Statistical analysis of the optimization algorithms

In this subsection, the TEP models are applied to evaluate the optimization capabilities of various algorithms on the Garver system. Table 2 presents the optimization results obtained from 20 runs, comprising metrics such as the best and worst fitnesses, average fitness, and computation time for each run. The results from model #1 demonstrate that all algorithms successfully obtained the minimum cost value of 556 million USD. Among its counterparts in model #1, KOA demonstrated the minimum average value, followed by WSO and TTAO, respectively. This establishes KOA as a competitive algorithm in the optimization of the Garver system.

**Table 2:** Optimization results of the optimization algorithms for the Garver system.

TEP model	Measure	SCHO	WO	SGA	TTAO	EEFO	KOA	DBO	SHO	SRS	WSO
O.F1	Best	556.00	556.00	556.00	556.00	556.00	556.00	556.00	556.00	556.00	556.00
	Worst	798.04	717.00	1007.0	617.00	690.00	586.00	931.00	919.00	677.75	604.00
	Average	614.72	586.80	753.30	571.59	601.05	559.51	650.20	700.50	632.14	567.45
	Time (s)	8.77	8.84	8.21	9.54	8.55	8.23	7.63	9.10	7.43	6.44
O.F2	Best	536.88	506.71	548.05	486.65	506.91	486.65	486.64	536.42	548.05	506.83
	Worst	699.15	584.71	894.81	556.32	670.91	506.88	816.38	908.96	697.00	585.10
	Average	583.17	538.10	710.01	520.07	536.61	500.78	623.74	646.68	627.96	535.36
	Time (s)	12.17	12.05	11.44	12.34	11.72	11.35	10.84	12.22	10.35	8.94
O.F3	Best	536.90	487.28	625.16	487.28	507.27	487.28	487.28	566.75	600.73	507.88
	Worst	993.43	598.14	873.69	766.26	637.29	537.47	779.42	963.02	795.13	852.92
	Average	778.55	543.58	782.34	581.00	562.89	506.76	692.77	752.1831	642.24	616.61
	Time (s)	13.9	13.81	13.47	14.02	13.25	13.17	12.65	13.84	11.71	10.12

In model #2, KOA, DBO, and TTAO demonstrated their efficiency in obtaining the best solutions at 486.6 million USD. However, KOA excelled in terms of the best average value over the executed runs. The average value of KOA was approximately 19.3 and 123 million USD units lower than that of TTAO and DBO, respectively, representing a reduction of about 3.7% and 19.7%, respectively.

When the planning model was expanded to incorporate FCL's planning model (model #3), among other algorithms, KOA, WO, TTAO, and DBO were identified as the best algorithms for determining the optimal solutions. However, KOA outperformed all other algorithms in obtaining the best average value, as shown in Table 2. The best solution and average value were approximately 487.28 and 506.76 million USD, respectively. Regarding the acquisition of the best average values, WO ranked as the second-best algorithm, followed by EEFO and TTAO, respectively.

**Figure 5:**
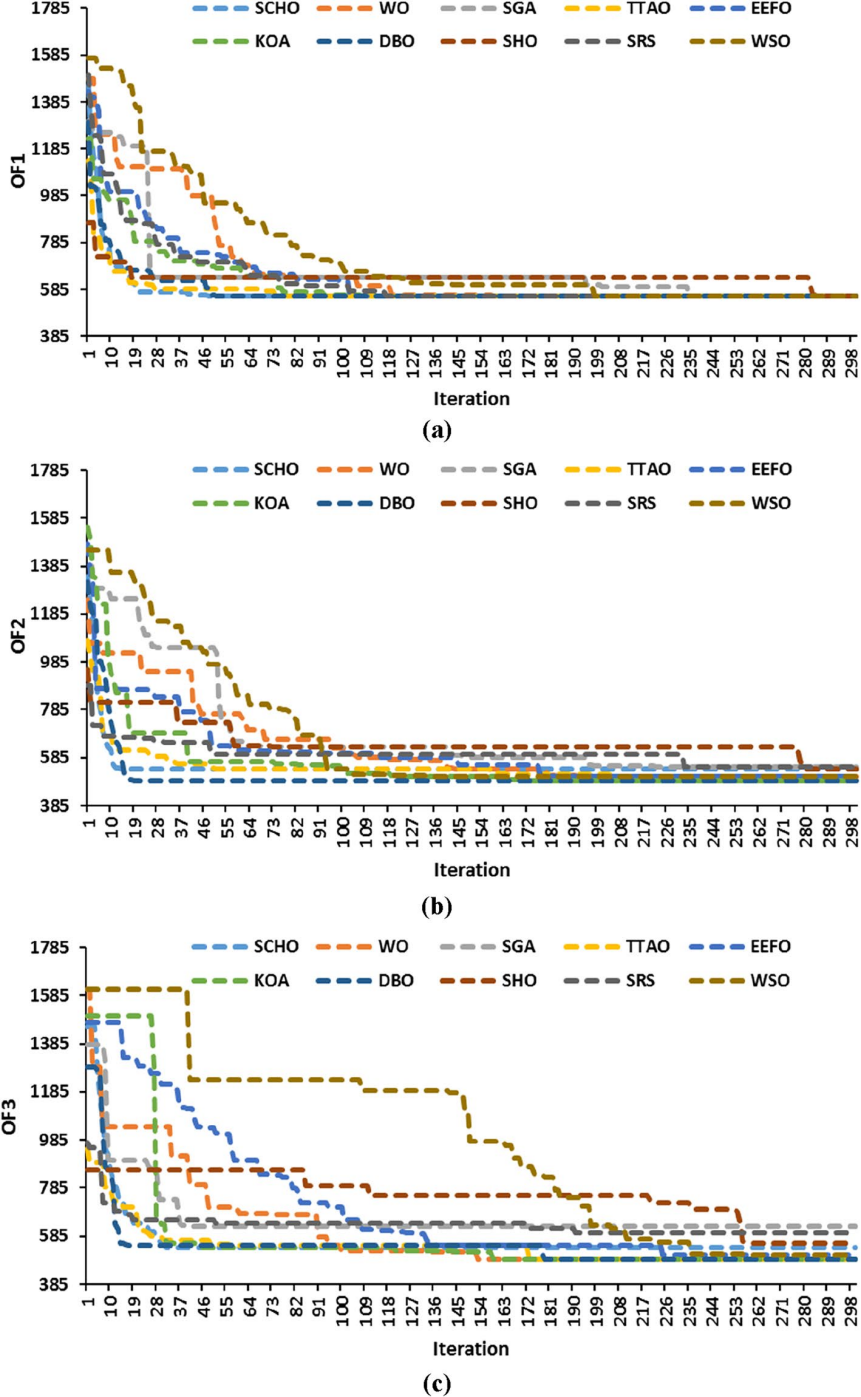
Convergence curve of the optimization algorithms for the Garver system: (**a**) model #1, (**b**) model #2, and (**c**) model #3.

The time values presented in Table 2 represent the average duration obtained from conducting 20 distinct runs. Figure 5 illustrates the convergence curves of all algorithms concerning the best achieved score so far. While all algorithms achieved convergence, WSO exhibited the most rapid convergence rate. Despite WSO demonstrating the quickest iteration, its accuracy falls below that of KOA, DBO, and TTAO, as corroborated by the data amalgamated in Table 2. SRS, SCHO, WSO, SHO, SGA, and EEFO exhibit stagnation at local extremes, especially when applied to solve model #2 and model #3, affirming the effectiveness of the exploitation phase of KOA, DBO, and TTAO, which demonstrates reliable exploration potential.

**Figure 6:**
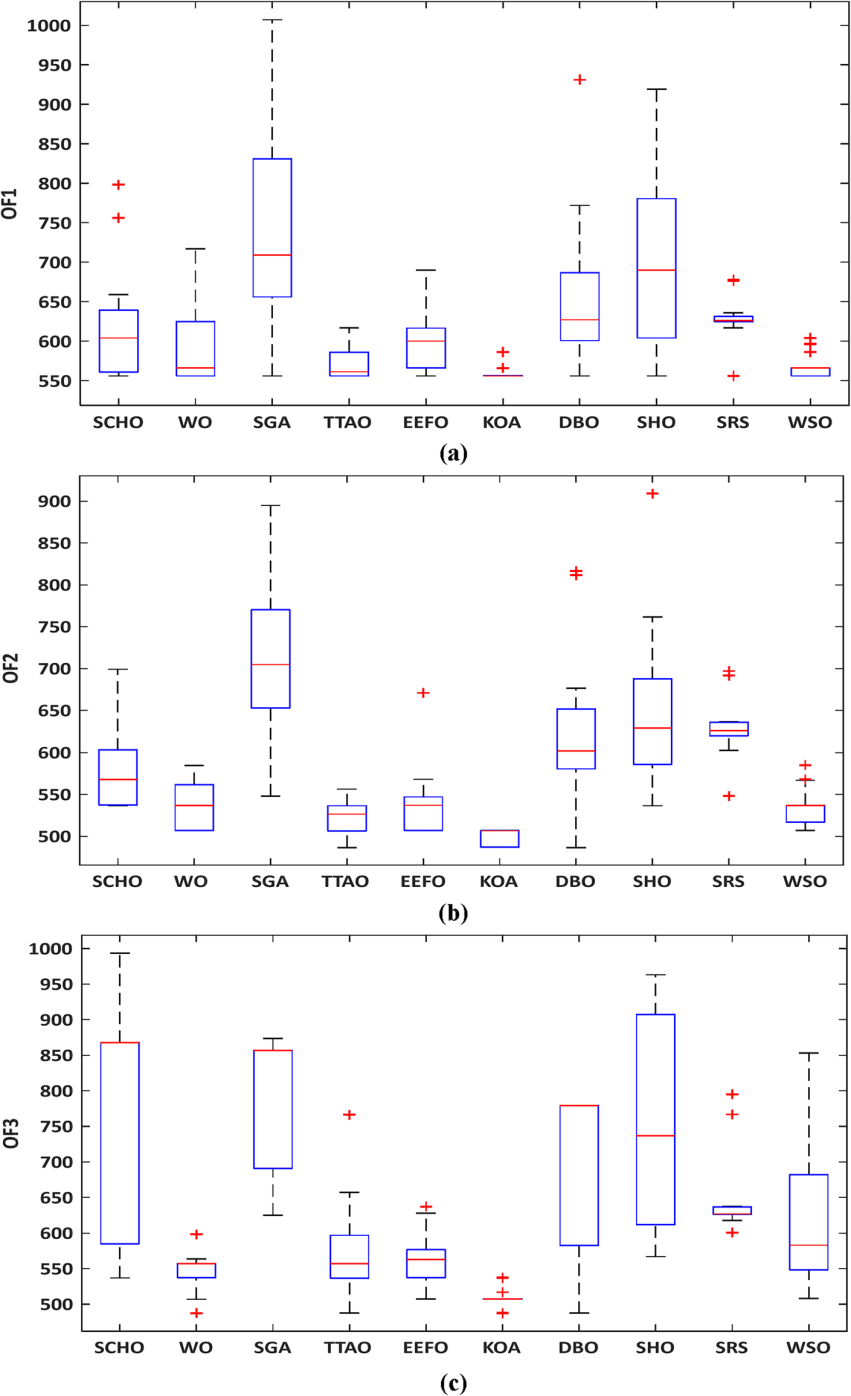
Variations’ box chart of runs for the Garver network: (**a**) model #1, (**b**) model #2, and (**c**) model #3.

In Fig. 6, a box plot illustrating the performance of the algorithms is presented. KOA stands out prominently, as evidenced by the smallest interquartile range displayed in the plot. Moreover, KOA attains the lowest worst objective value over the three models, outperforming other algorithms.

The result gathered from the Wilcoxon rank sum test provides a crucial metric known as the p-value, determining the significance of the evaluated algorithm's superiority over its competitors. In this analysis, an algorithm achieves statistical significance if its p-value is below 0.05. Table 3 presents the results of the Wilcoxon rank sum test. Complemented by data from 20 simulation runs, the symbols “+”, and “−” denote whether the algorithms achieve statistical significance or not, respectively. The results supported the data provided in Table 2, showing that KOA delivered the best performance in solving Model #1. Additionally, KOA, DBO, and TTAO consistently outperformed alternative algorithms, particularly in Model #2. For Model #3, the Wilcoxon rank sum test results confirmed the efficiency of KOA, WO, TTAO, and DBO compared to all other algorithms.

**Table 3:** Wilcoxon rank sum test between the algorithms for the Garver system.

Algorithm	TEP model	Measure	SCHO	WO	SGA	TTAO	EEFO	KOA	DBO	SHO	SRS	WSO
SCHO	O.F1	*p-*value	NA	0.04102	0.00012	7.11E−03	0.59784	3.71E−05	0.18941	0.0143	6.20E−02	0.0008
		*H*	NA	+	+	+	−	+	−	+	+	+
	O.F2	*p-*value	NA	0.0004	2.60E−05	1.92E−07	0.0007	6.80E-08	0.0385	0.017	5.12E−03	0.000104
		*H*	NA	+	+	+	+	+	+	+	+	+
	O.F3	*p-*value	NA	9.25E−07	1.42E−01	3.21E−05	2.53E−05	6.24E−08	0.010812	0.712296	3.46E−02	0.001358
		*H*	NA	+	−	+	+	+	+	−	+	+
WO	O.F1	*p-*value	0.04102	NA	3.04E−06	6.85E−01	0.16724	7.97E−04	0.0121	0.0002	4.13E−04	0.6257
		*H*	+	NA	+	−	−	+	+	+	+	−
	O.F2	*p-*value	0.0004	NA	1.66E−07	2.56E-03	0.4734	7.95E−07	3.71E−05	5.87E−06	1.66E−07	0.473481
		*H*	+	NA	+	+	−	+	+	+	+	−
	O.F3	*p-*value	9.25E−07	NA	4.94E−08	3.29E−01	0.063195	3.75E−05	0.000111	1.08E−07	6.28E−08	0.000602
		*H*	+	NA	+	−	−	+	+	+	+	+
SGA	O.F1	*p-*value	0.00012	3.04E−06	NA	1.20E−06	9.74E−06	1.20E−06	0.00332	1.81E−01	0.0001	4.51E−07
		*H*	+	+	NA	+	+	+	+	+	+	+
	O.F2	*p-*value	2.60E−05	1.66E−07	NA	7.90E−08	3.42E−07	6.80E−08	2.14E−03	2.75E−02	0.002	1.23E−07
		*H*	+	+	NA	+	+	+	+	+	+	+
	O.F3	*p-*value	1.42E−01	4.94E−08	NA	9.89E−07	7.28E−08	5.37E−08	1.72E−02	3.46E−01	5.65E−06	1.76E−05
		*H*	−	−	NA	+	+	+	+	−	+	+
TTAO	O.F1	*p-*value	7.11E-03	6.85E−01	1.20E−06	NA	2.31E−02	0.45695	1.01E−03	2.04E−05	1.20E−06	1.10E−01
		*H*	+	−	+	NA	+	−	+	+	+	−
	O.F2	*p-*value	1.92E−07	2.56E−03	7.90E−08	NA	9.79E−03	1.44E−02	2.36E−06	1.66E−07	7.90E−08	1.12E−03
		*H*	+	+	+	NA	+	+	+	+	+	+
	O.F3	*p-*value	3.21E−05	3.29E−03	9.89E−07	NA	9.68E−03	4.83E−06	1.67E−03	5.08E−05	3.75E−04	1.02E−04
		*H*	+	+	+	NA	+	+	+	+	+	+
EEFO	O.F1	*p-*value	0.59784	0.16724	9.74E−06	2.31E−02	NA	5.09E−04	0.04366	0.00148	6.22E−04	0.0020
		*H*	−	−	+	+	NA	+	+	+	+	+
	O.F2	*p-*value	0.0007	0.4734	3.42E−07	9.79E−03	NA	6.80E−08	4.17E−05	1.25E−05	1.38E−06	0.655
		*H*	+	−	+	+	NA	+	+	+	+	-
	O.F3	*p-*value	2.53E−05	0.063195	7.28E−08	9.68E−03	NA	6.92E−07	0.000989	4.95E−06	5.87E−06	0.027483
		*H*	+	−	+	+	NA	+	+	+	+	+
KOA	O.F1	*p-*value	3.71E−05	7.97E−04	1.20E−06	0.45695	5.09E−04	NA	2.21E−04	1.58E−06	1.20E−06	9.89E−03
		*H*	+	+	+	−	+	NA	+	+	+	+
	O.F2	*p-*value	6.80E−08	7.95E−07	6.80E−08	1.44E−02	6.80E−08	NA	1.20E−06	6.80E−08	6.80E−08	1.23E−07
		*H*	+	+	+	+	+	NA	+	+	+	+
	O.F3	*p-*value	6.24E−08	3.75E−05	5.37E−08	4.83E−06	6.92E−07	NA	1.08E−05	6.41E−08	6.80E−08	1.66E−07
		*H*	+	+	+	+	+	NA	+	+	+	+
DBO	O.F1	*p-*value	0.18941	0.0121	0.00332	1.01E−03	0.04366	2.21E−04	NA	0.18045	9.03E−01	0.0004
		*H*	−	+	+	+	+	+	NA	+	−	+
	O.F2	*p-*value	0.0385	3.71E−05	2.14E−03	2.36E−06	4.17E−05	1.20E−06	NA	0.0350	3.94E−04	2.04E−05
		*H*	+	+	+	+	+	+	NA	+	+	+
	O.F3	*p-*value	0.010812	0.000111	1.72E−02	1.67E−03	0.000989	1.08E−05	NA	0.0411583	2.24E−03	0.025901
		*H*	+	+	+	+	+	+	NA	+	+	
SHO	O.F1	*p-*value	0.01436	0.00024	1.81E−01	2.04E−05	0.00148	1.58E−06	0.18045	NA	1.48E−01	3.05E−06
		*H*	+	+	−	+	+	+	−	NA	+	+
	O.F2	*p-*value	0.0179	5.87E−06	2.75E−02	1.66E−07	1.25E−05	6.80E−08	0.03507	NA	7.97E−01	5.87E−06
		*H*	+	+	+	+	+	+	+	NA	−	+
	O.F3	*p-*value	0.712296	1.08E−07	3.46E−01	5.08E−05	4.95E−06	6.41E−08	0.0411583	NA	7.15E−03	1.32E−03
		*H*	−	+	−	+	+	+	+	NA	+	+
SRS	O.F1	*p-*value	6.20E−02	4.13E−04	0.00014	1.20E−06	6.22E−04	1.20E−06	9.03E−01	1.48E−01	NA	1.19E−06
		*H*	−	+	+	+	+	+	−	−	NA	+
	O.F2	*p-*value	5.12E−03	1.66E−07	0.0020	7.90E−08	1.38E−06	6.80E−08	3.94E−04	7.97E−01	NA	1.23E−07
		*H*	+	+	+	+	+	+	+	−	NA	+
	O.F3	*p-*value	3.46E−02	6.28E−08	5.65E−06	3.75E−04	5.87E−06	6.80E−08	2.24E−03	7.15E−03	NA	1.20E−01
		*H*	+	+	+	+	+	+	−	+	NA	−
WSO	O.F1	*p-*value	0.00083	0.62577	4.51E−07	1.10E−01	0.00203	9.89E−03	0.00047	3.05E−06	1.19E−06	NA
		*H*	+	−	+	−	+	+	+	+	+	NA
	O.F2	*p-*value	0.0001	0.4734	1.23E−07	1.12E−03	0.6553	1.23E−07	2.04E−05	5.87E−06	1.23E−07	NA
		*H*	+	−	+	+	−	+	+	+	+	NA
	O.F3	*p-*value	0.001358	0.000602	1.76E−05	1.02E−03	0.027483	1.66E−07	0.025901	1.32E−03	1.20E−01	NA
		*H*	+	+	+	+	+	+	+	+	−	NA

#### The Garver network configuration

Table 4 outlines the incorporation of new components necessary to expand the Garver network in order to meet electrical demand. These components are selected from the best-performing runs in each model. As indicated in Table 4, in model #1, five circuits are crucial for supplying the loads, located along routes 2–3, 3–5, and 4–6. In model #2, the integration of the TCSCS planning model into the TEP model reduces the number of installed circuits from 5 to 2, consequently lowering the overall planning cost. Furthermore, Table 4 highlights the significance of installing FCLs to restrict short-circuit currents to below 6.5 p.u.

**Table 4:** Installed projects required for the Garver system.

TEP model	Added circuits	No. of added TCSCs	No. added FCLs
Model #1	2–3 (1); 3–5(1); 4–6 (3)	–	–
Model #2	4–6 (2)	6	–
Model #32	4–6 (2)	6	3

### The WDN

#### Statistical analysis of the optimization algorithms

In this subsection, the performance of the algorithms is evaluated on the WDN. Each algorithm undergoes 20 independent runs for each test, and the statistical findings are synthesized in Table 5. The results of model #1 showed that KOA, TTAO, and SHO succeeded in obtaining the best solution, valued at 401.22 million USD. Despite the increased scale of the system, KOA still provides the minimum average value. It was observed that KOA provided the best average value of 402.11 million USD, which was lower by about 17.44 million USD than SGA, representing the worst value.

**Table 5:** Optimization results of the optimization algorithms for the WDN.

TEP model	Measure	SCHO	WO	SGA	TTAO	EEFO	KOA	DBO	SHO	SRS	WSO
O.F1	Best	401.96	402.67	404.94	401.22	401.84	401.22	402.37	401.22	406.72	402.34
	Worst	416.02	410.12	434.92	407.11	416.98	405.46	420.07	412.07	423.21	414.92
	Average	408.69	406.59	419.55	402.78	408.03	402.11	407.76	405.94	418.05	407.56
	Time (s)	64.20	67.79	61.86	118.52	74.52	66.23	58.63	91.02	56.21	27.35
O.F2	Best	400.09	396.84	404.25	395.69	400.42	393.35	397.36	396.72	418.35	400.62
	Worst	411.38	407.72	438.28	402.45	410.64	400.39	412.56	406.82	429.92	413.58
	Average	404.72	401.92	421.79	398.20	405.30	395.81	404.39	402.97	424.56	406.94
	Time (s)	76.4	81.61	70.169	131.72	88.24	77.63	67.43	103.88	66.54	35.4
O.F3	Best	401.91	397.30	403.41	396.74	400.73	394.85	398.11	397.12	407.52	401.5497
	Worst	414.48	411.54	431.62	401.67	413.86	403.33	413.77	406.94	429.69	411.0904
	Average	406.71	402.89	414.10	397.98	406.60	396.45	404.72	401.87	422.38	406.5078
	Time (s)	81.04	85.06	75.63	134.05	92.89	81.84	70.22	105.17	70.05	38.33

In model #2, KOA continues to prove its efficiency in obtaining the best solutions as shown in Table 5. The lowest cost function was 393.35 million USD. TTAO is the second-best algorithm, followed by SHO, WO, and DBO, respectively. In terms of average values, KOA still provides the best value at 395.81 million USD, followed by TTAO and WO, respectively. The average value of KOA was lower by about 2.39 and 6.11 million USD for TTAO and WO, respectively.

When the planning model was expanded to incorporate the planning model of FCLs (model #3), among other algorithms, KOA emerged as the top-performing algorithm in determining optimal solutions and achieving the lowest average value, as depicted in Table 5. The optimal solution and average value stood at approximately 394.85 and 396.45 million USD, respectively. Following closely in terms of both optimal solution and average values, TTAO ranked as the second-best algorithm, trailed by SHO and WO, respectively. Their optimal solutions exceeded KOA's by approximately 1.89, 2.27, and 2.45 million USD, respectively, while their average values surpassed KOA's by about 1.53, 5.42, and 6.44 million USD, respectively.

**Figure 7:**
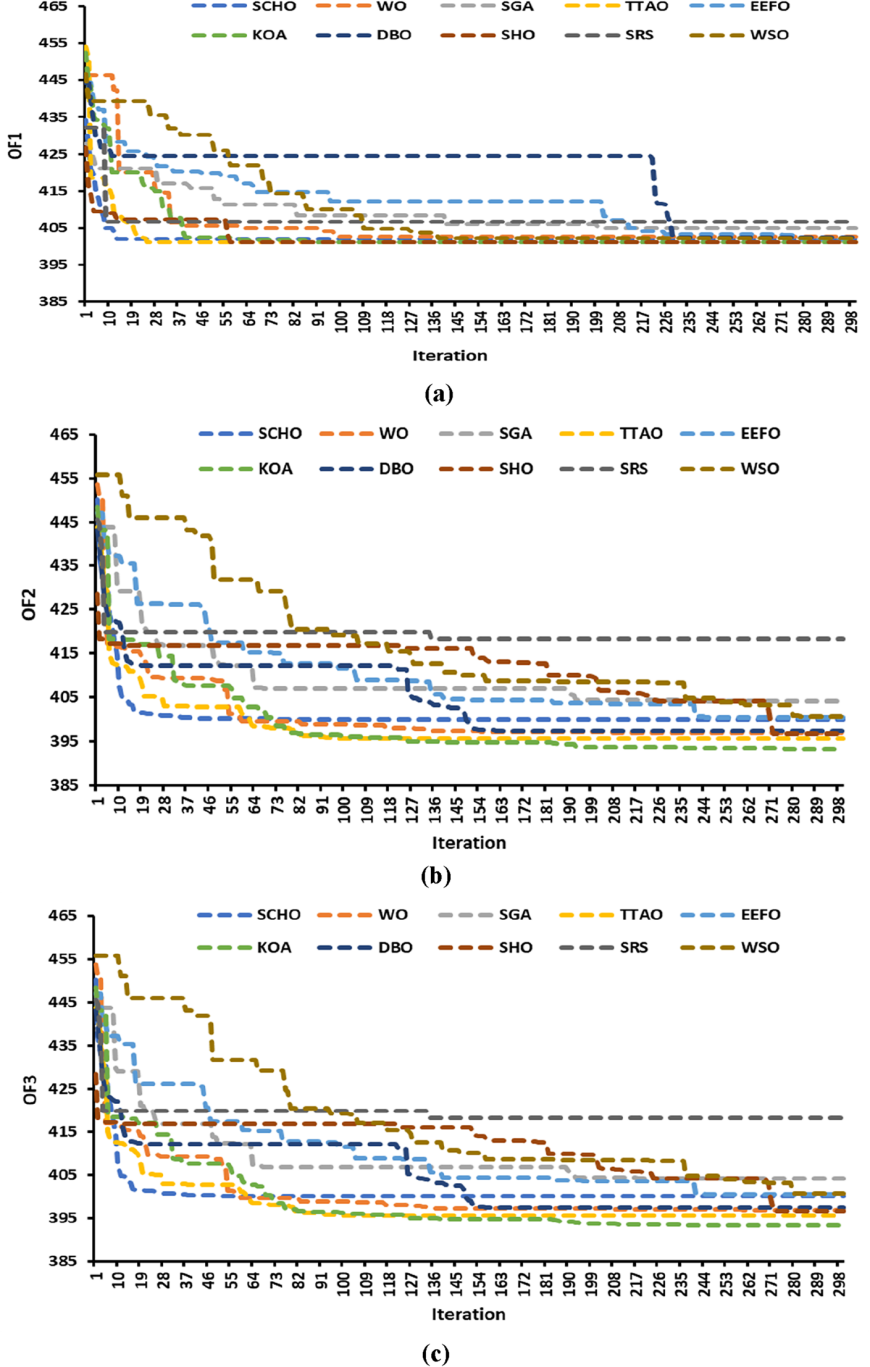
Convergence curve of the optimization algorithms for the WDN: (**a**) model #1, (**b**) model #2, and (**c**) model #3.

The data presented in Table 5 shows the average duration obtained by running the WDN for 20 iterations. Similar to the Garver network, it is apparent that six algorithms require less computational time than KOA, while the remaining algorithms (WO, TTAO, EEFO, SHO) take longer. Figure 7 displays the convergence curves of the best run for all the algorithms. Table 5 also highlights that the WSO also demonstrated the shortest computational times, approximately 2–3 times faster than those of other algorithms. Despite its rapid convergence, the average solution value obtained is higher compared to KOA, TTAO, and SHO. In TEP, prioritizing the quality of solutions is paramount over the speed of the algorithm.

**Figure 8:**
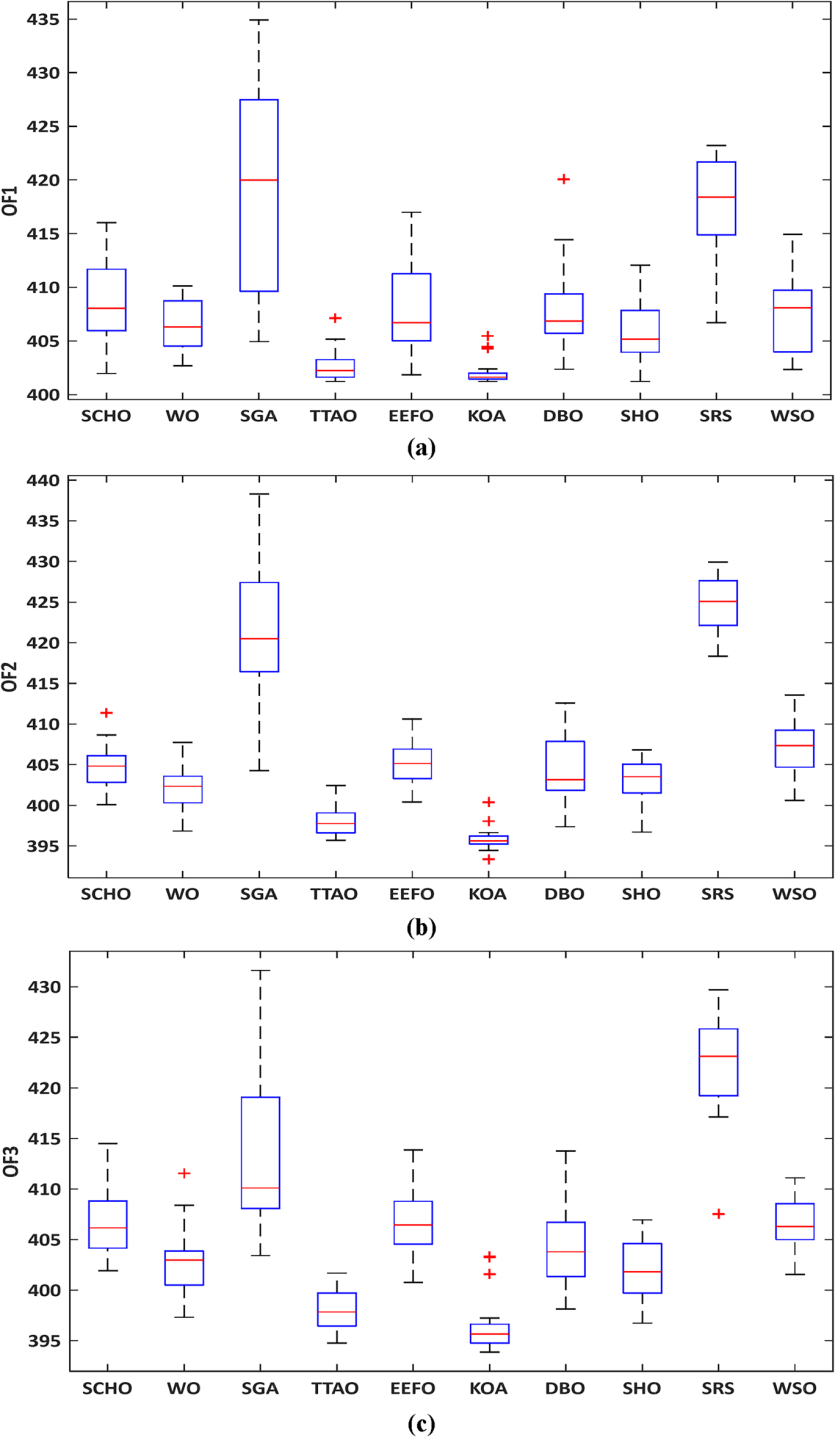
Variations’ box chart of runs for the WDN: (**a**) model #1, (**b**) model #2, and (**c**) model #3.

The performance of the algorithms is depicted in Fig. 8 through a box plot, offering a comprehensive visual representation of their comparative efficacy. Remarkably, KOA emerges as the leading performer, characterized by the smallest interquartile range observed in the plot. This narrow range signifies a more consistent performance across different scenarios, reflecting the algorithm's robustness and reliability. Moreover, KOA achieves the lowest worst objective value among all algorithms evaluated, underscoring its exceptional capability in finding optimal solutions even under challenging conditions. This standout performance further solidifies KOA's position as a promising algorithm for addressing complex optimization problems such as TEP.

The Wilcoxon rank sum test results are presented in Table 6, providing a detailed comparison of the performance of different algorithms. The symbols “+”, and “−” denote whether the algorithms achieve statistical significance or not, respectively. The results supported the data provided in Table 5, showing that KOA delivered the best performance in solving across all tested models. The results indicate that the KOA algorithm demonstrates superior performance, consistent with the statistical analyses conducted. Notably, the KOA algorithm shows exceptional effectiveness in tackling TEP issues, as evidenced by its consistently high performance across different models. These findings suggest that the KOA algorithm could be a suitable choice for addressing TEP problems, given its superior performance in terms of accuracy and reliability.

**Table 6:** Wilcoxon rank-sum test between the algorithms for the WDN.

Algorithm	TEP model	Measure	SCHO	WO	SGA	TTAO	EEFO	KOA	DBO	SHO	SRS	WSO
SCHO	O.F1	*p-*value	NA	0.123	0.00041	5.46E−06	0.542	5.42E−07	0.3793	0.0273	3.07E−06	0.440
		*H*	NA	−	+	+	−	+	−	+	+	−
	O.F2	*p-*value	NA	0.00655	3.42E−07	2.96E−07	0.56085	7.90E−08	0.65536	0.11985	6.80E−08	0.0256
		*H*	NA	+	+	+	−	+	−	−	+	+
	O.F3	*p-*value	NA	0.000305	2.14E−03	6.80E−08	0.924573	9.17E−08	0.053103	0.000144	2.22E−07	0.946084
		*H*	NA	+	+	+	−	+	−	+	+	−
WO	O.F1	*p-*value	0.123091	NA	3.71E−05	7.06E−06	0.273285	7.74E−07	0.456951	0.35025	2.56E−07	0.516168
		*H*	−	NA	+	+	−	+	−	−	+	−
	O.F2	*p-*value	0.00655	NA	1.06E−07	1.61E−04	0.00162	1.92E−07	0.0909	0.15557	6.80E−08	0.0001
		*H*	+	NA	+	+	+	+	−	−	+	+
	O.F3	*p-*value	0.00030	NA	2.36E−06	3.99E−06	0.00101	5.17E−06	0.23932	0.35070	9.17E−08	0.0002
		*H*	+		+	+	+	+	−	−	+	+
SGA	O.F1	*p-*value	0.000416	3.71E−05	NA	1.14E−07	0.000179	8.90E−08	0.000144	1.86E−05	0.635945	0.000104
		*H*	+	+	NA	+	+	+	+	+	−	+
	O.F2	*p-*value	3.42E−07	1.06E−07	NA	6.80E−08	4.54E−07	6.80E−08	2.56E−07	2.22E−07	0.096	7.95E−07
		*H*	+	+	NA	+	+	+	+	+	−	+
	O.F3	*p-*value	2.14E−03	2.36E−06	NA	6.80E−08	2.34E−03	6.80E−08	3.38E−04	4.54E−07	0.007	9.21E−04
		*H*	+	+	NA	+	+	+	+	+	+	+
TTAO	O.F1	*p-*value	5.47E−06	7.06E−06	1.14E−07	NA	6.63E−06	0.03719	3.71E−06	0.00013	7.83E−08	2.58E−05
		*H*	+	+	+	NA	+	+	+	+	+	+
	O.F2	*p-*value	2.96E−07	1.61E−04	6.80E−08	NA	3.42E−07	2.92E−05	5.87E−06	8.60E−06	6.80E−08	1.66E−07
		*H*	+	+	+	NA	+	+	+	+	+	+
	O.F3	*p-*value	6.80E−08	3.99E−06	6.80E−08	NA	9.17E−08	3.34E−03	1.38E−06	1.44E−04	6.80E−08	7.90E−08
		*H*	+	+	+	NA	+	+	+	+	+	+
EEFO	O.F1	*p-*value	0.5427	0.2732	0.00017	6.63E−06	NA	8.89E−07	0.839232	0.1132	1.20E−06	0.675
		*H*	−	−	+	+	NA	+	−	−	+	−
	O.F2	*p-*value	0.56085	0.00162	4.54E−07	3.42E−07	NA	6.80E−08	0.32348	0.0239	6.80E−08	0.0909
		*H*	−	+	+	+	NA	+	−	+	+	−
	O.F3	*p-*value	0.92457	0.00101	2.34E−03	9.17E−08	NA	2.22E−07	0.09090	0.00024	1.66E−07	0.8817
		*H*	−	−	+	+	NA	+	−	+	+	−
KOA	O.F1	*p-*value	5.42E−07	7.74E−07	8.90E−08	0.037195	8.89E−07	NA	3.56E−07	1.44E−05	6.59E−08	1.54E−06
		*H*	+	+	+	+	+	NA	+	+	+	+
	O.F2	*p-*value	7.90E−08	1.92E−07	6.80E−08	2.92E−05	6.80E−08	NA	1.23E−07	1.43E−07	6.80E−08	6.80E−08
		*H*	+	+	+	+	+	NA	+	+	+	+
	O.F3	*p-*value	9.17E−08	5.17E−06	6.80E−08	3.34E−03	2.22E−07	NA	2.06E−06	1.41E−05	6.80E−08	1.43E−07
		*H*	+	+	+	+	+		+	+	+	+
DBO	O.F1	*p-*value	0.37933	0.45695	0.00014	3.71E−06	0.83923	3.56E−07	NA	0.10714	2.36E−06	0.9892
		*H*	−	−	+	+	−	+	NA	−	+	−
	O.F2	*p-*value	0.65536	0.09090	2.56E−07	5.87E−06	0.32348	1.23E−07	NA	0.49033	6.80E−08	0.063
		*H*	−	−	+	+	−	+	NA	−	+	−
	O.F3	*p-*value	0.05310	0.23932	3.38E−04	1.38E−06	0.09090	2.06E−06	NA	0.06389	1.23E−07	0.0565
		*H*	−	−	+	+	−	+	NA	−	+	−
SHO	O.F1	*p-*value	0.027338	0.35025	1.86E−05	0.000132	0.113213	1.44E−05	0.107148	NA	2.16E−07	0.2179
		*H*	+	−	+	+	−	+	−	NA	+	−
	O.F2	*p-*value	0.11985	0.15557	2.22E-07	8.60E-06	0.02390	1.43E-07	0.49033	NA	6.80E-08	0.0003
		*H*	−	−	+	+	+	+	−	NA	+	+
	O.F3	*p-*value	0.00014	0.35070	4.54E-07	1.44E-04	0.00024	1.41E-05	0.06389	NA	6.80E-08	9.28E-05
		*H*	+	−	+	+	+	+	−	NA	+	+
SRS	O.F1	*p-*value	3.07E-06	2.56E-07	0.635945	7.83E-08	1.20E-06	6.59E-08	2.36E-06	2.16E-07	NA	9.12E-07
		*H*	+	+	−	+	+	+	+	+	NA	+
	O.F2	*p-*value	6.80E-08	6.80E-08	0.09619	6.80E-08	6.80E−08	6.80E−08	6.80E−08	6.80E−08	NA	6.80E−08
		*H*	+	+	−	+	+	+	+	+	NA	+
	O.F3	*p-*value	2.22E−07	9.17E−08	0.00711	6.80E−08	1.66E−07	6.80E−08	1.23E−07	6.80E−08	NA	1.66E−07
		*H*	+	+	+	+	+	+	+	+	NA	+
WSO	O.F1	*p-*value	0.440728	0.516168	0.000104	2.58E−05	0.675	1.54E−06	0.989208	0.217952	9.12E−07	NA
		*H*	−	−	+	+	−	+	−	−	+	NA
	O.F2	*p-*value	0.02563	0.00010	7.95E−07	1.66E−07	0.09090	6.80E−08	0.06389	0.00037	6.80E−08	NA
		*H*	+	+	+	+	−	+	−	+	+	NA
	O.F3	*p-*value	0.94608	0.00024	9.21E−04	7.90E−08	0.88173	1.43E−07	0.05651	9.28E−05	1.66E−07	NA
		*H*	−	+	+	+	−	+	−	+	+	NA

#### The WDN configuration

Table 7 gives a summary of the new components that are required to expand the WDN. These components are chosen from the best-performing runs in each model. As shown in Table 7, in model #1, seven circuits are required to supply the loads that are located along routes 5–6, 33–53, 5–53, 36–53, and 20–53. In model #2, the incorporation of the TCSCS planning model into the TEP model reduces the number of installed circuits from 7 to 4, resulting in a decrease in the overall planning cost. Additionally, Table 7 highlights the significance of installing four FCLs to limit short-circuit currents to below 9 p.u.

**Table 7:** Installed projects required for the WDN.

TEP model	Added circuits	No. of added TCSCs	No. added FCLs
Model #1	5–6 (1); 33–53 (1); 5–53(2); 36–53 (2); 20–53 (1)	–	–
Model #2	6–34 (1); 5–53 (1); 36–53 (2)	6	–
Model #32	6–34 (1); 5–53 (1); 36–53 (2)	6	4

### The IEEE 118-bus system

#### Statistical analysis of the optimization algorithms

Table 8 presents the optimization results. In Model #1, all algorithms except SRS and WSO achieved the minimum cost value of 348.62 million USD. Notably, KOA demonstrated the lowest average value among the algorithms, followed by SHO and WO. KOA’s average cost was 348.81 million USD, which is approximately 2.74% and 3.63% lower than those of SHO and WO, respectively.

**Table 8:** Optimization results of the optimization algorithms for the IEEE 118-bus system.

TEP model	Measure	SCHO	WO	SGA	TTAO	EEFO	KOA	DBO	SHO	SRS	WSO
O.F1	Best	348.62	348.62	348.62	348.62	348.62	348.62	348.62	348.62	358.52	364.92
	Worst	387.72	383.12	670.92	604.02	374.82	349.34	578.72	374.82	474.82	469.924
	Average	364.14	361.93	522.81	463.11	361.07	348.81	464.53	358.63412	412.44	443.5401
	Time (s)	120.45	128.21	116.45	226.5	142.31	127.02	110.7	172.79	107.02	77.21
O.F2	Best	354.01	351.52	333.96	338.60	353.20	333.96	333.96	333.96	362.20	361.46
	Worst	394.17	355.14	648.58	710.94	354.38	345.01	783.26	369.77	672.70	719.43
	Average	362.68	353.03	390.13	493.26	353.92	340.38	410.55	345.74	502.25	490.23
	Time (s)	132.02	1.38.4	124.66	239.21	154.21	132.4	119.45	184.03	112.33	80.11
O.F3	Best	355.25	351.97	337.03	367.43	355.48	337.03	337.03	338.57	341.12	341.12
	Worst	376.94	359.40	1807.6	649.23	356.18	347.71	941.11	377.92	367.83	365.20
	Average	364.18	354.78	551.15	487.94	355.84	341.98	422.80	351.33	353.60	351.51
	Time (s)	141.56	147.35	133.07	256.56	163.45	144.03	124.89	195.12	119.32	85.9

In Model #2, KOA, DBO, SGA, and SHO were effective in reaching the optimal solution of 333.96 million USD. However, KOA distinguished itself by achieving the best average value across all runs. KOA’s average was approximately 5.36 million USD lower than that of SHO, which ranked second, representing a reduction of about 1.55%.

In Model #3, KOA, SGA, and DBO remained effective in obtaining the optimal solution of 337.03 million USD. Among these, KOA excelled, achieving the best average value, as shown in Table 8. The optimal average value was approximately 341.98 million USD, which is 2.66% lower than that of SHO, which obtained the second-best value.

**Figure 9:**
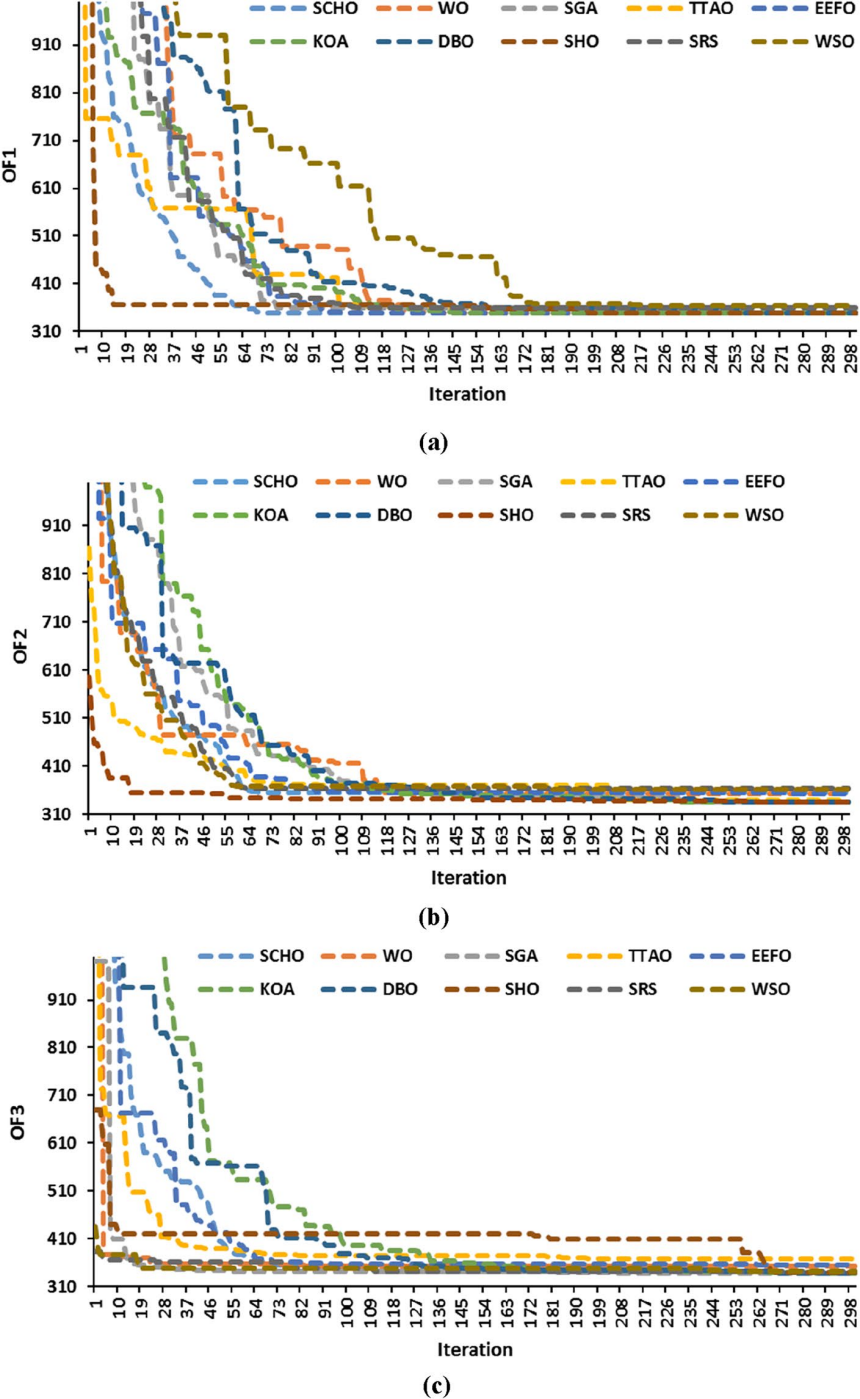
Convergence curve of the optimization algorithms for the IEEE 118-bus system: (**a**) model #1, (**b**) model #2, and (**c**) model #3.

**Figure 10:**
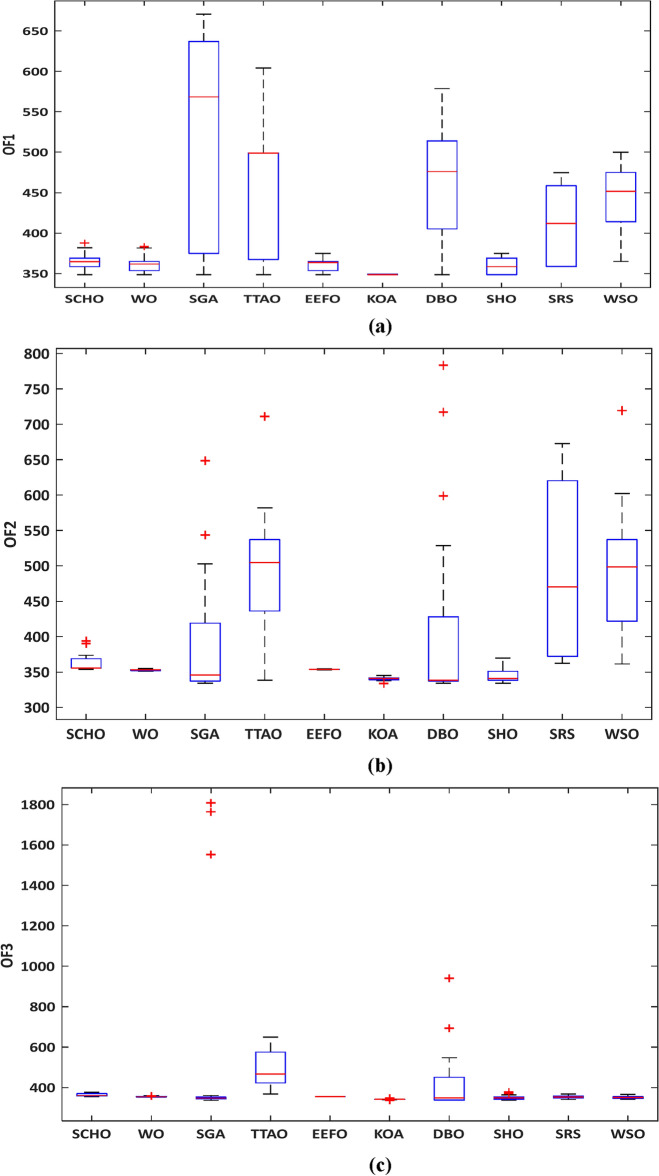
Variations’ box chart of runs for the IEEE 118-bus system: (**a**) model #1, (**b**) model #2, and (**c**) model #3.

Figure 9 illustrates the convergence curves of all algorithms concerning the best achieved score to date. While all algorithms converged, WSO exhibited the most rapid convergence rate. Figure 10 presents a box plot comparing the performance of the algorithms. KOA stands out prominently, evidenced by the smallest interquartile range displayed in the plot.

Table 9 presents the results of the Wilcoxon rank-sum test, based on data from 20 simulation runs. The results confirm the efficiency of KOA, SGA, SHO, and DBO in system planning. However, KOA emerged as the superior algorithm.

**Table 9:** Wilcoxon rank sum test between the algorithms for the IEEE 118-bus system.

Algorithm	TEP model	Measure	SCHO	WO	SGA	TTAO	EEFO	KOA	DBO	SHO	SRS	WSO
SCHO	O.F1	*p-*value	NA	0.1799	0.0001	7.42E−03	0.2274	7.58E−06	2.30E−05	0.027	4.67E−02	5.23E−07
		*H*	NA	−	+	+	−	+	+	+	+	+
	O.F2	*p-*value	NA	1.23E−07	7.11E−03	1.58E−06	2.96E−07	6.80E−08	0.0071	2.30E−05	4.36E−06	5.23E−07
		*H*	NA	+	+	+	+	+	+	+	+	+
	O.F3	*p-*value	NA	2.06E−06	4.60E−04	2.21E−07	1.25E−05	6.80E−08	0.394171	0.000275	1.79E−04	1.05E−06
		*H*	NA	+	+	+	+	+	−	+	+	+
WO	O.F1	*p-*value	0.1799	NA	2.10E−05	5.45E−03	0.8051	7.03E−03	7.82E−06	0.967	2.51E−03	1.98E−07
		*H*	−	NA	+	+	−	+	+	-	+	+
	O.F2	*p-*value	1.23E−07	NA	7.11E−03	1.20E−06	0.0008	6.80E−08	0.0071	0.001	6.53E−08	6.80E−08
		*H*	+	NA	+	+	+	+	+	+	+	+
	O.F3	*p-*value	2.06E−06	NA	2.80E−03	6.79E−08	0.0114	6.80E−08	0.6359	0.004	3.23E−01	0.007
		*H*	+	NA	+	+	+	+	−	+	−	+
SGA	O.F1	*p-*value	0.0001	2.10E−05	NA	4.55E−02	1.40E−05	1.20E−06	0.1165	1.49E−05	0.007	0.147
		*H*	+	+	NA	+	+	+	−	−	+	−
	O.F2	*p-*value	7.11E−03	7.11E−03	NA	5.09E−04	7.11E−03	5.48E−03	3.72E−01	4.90E−01	0.0001	2.47E−04
		*H*	+	+	NA	+	+	+	−	−	+	+
	O.F3	*p-*value	4.60E−04	2.80E−03	NA	1.61E−04	1.23E−03	4.96E−05	8.29E−01	7.97E−01	0.107511	2.29E−01
		*H*	+	+	NA	+	+	+	−	−	−	−
TTAO	O.F1	*p-*value	7.42E−03	5.45E−03	4.55E−02	NA	2.13E−03	0.0068	8.38E−01	0.006	3.06E−02	1.41E−01
		*H*	+	+	+	NA	+	+	−	+	+	−
	O.F2	*p-*value	1.58E−06	1.20E−06	5.09E−04	NA	1.20E−06	6.92E−07	2.92E−03	5.23E−07	9.46E−01	9.03E−01
		*H*	+	+	+	NA	+	+	+	+	−	-
	O.F3	*p-*value	2.21E−07	6.79E−08	1.61E−04	NA	6.79E−08	6.79E−08	3.06E−03	1.06E−07	7.89E−08	6.79E−08
		*H*	+	+	+	NA	+	+	+	+	+	+
EEFO	O.F1	*p-*value	0.2274	0.8051	1.40E−05	2.13E−03	NA	6.96E−03	7.53E−06	0.902	1.63E−02	9.86E−08
		*H*	−	−	+	+	NA	+	+	−	+	+
	O.F2	*p-*value	2.96E−07	0.0008	7.11E−03	1.20E−06	NA	6.80E−08	0.0071	0.000275	6.53E−08	6.80E−08
		*H*	+	+	+	+	NA	+	+	+	+	+
	O.F3	*p-*value	1.25E−05	0.0114	1.23E−03	6.79E−08	NA	6.80E−08	0.5978	0.001	1.08E−01	0.000161
		*H*	+	+	+	+	NA	+	−	+	−	+
KOA	O.F1	*p-*value	7.58E−06	7.03E−03	1.20E−06	0.0068	6.96E−03	NA	1.20E−06	5.98E−04	6.69E−08	6.80E−08
		*H*	+	+	+	+	+	NA	+	+	+	+
	O.F2	*p-*value	6.80E−08	6.80E−08	5.48E−03	6.92E−07	6.80E−08	NA	2.13E−04	4.33E−04	6.53E−08	6.80E−08
		*H*	+	+	+	+	+	NA	+	+	+	
	O.F3	*p-*value	6.80E−08	6.80E−08	4.96E−05	6.79E−08	6.80E−08	NA	2.04E−03	2.47E−04	1.38E−06	1.58E−06
		*H*	+	+	+	+	+	NA	+	+	+	+
DBO	O.F1	*p-*value	2.30E−05	7.82E−06	0.1165	8.38E−01	7.53E−06	1.20E−06	NA	9.75E−06	2.07E−02	0.285
		*H*	+	+	−	−	+	+	NA	+	+	−
	O.F2	*p-*value	0.0071	0.0071	3.72E−01	2.92E−03	0.0071	2.13E−04	NA	0.516	9.07E−04	0.001
		*H*	+	+	−	+	+	+	NA	−	+	+
	O.F3	*p-*value	0.3941	0.6359	8.29E−01	3.06E−03	0.5978	2.04E−03	NA	0.755	9.25E−01	0.967
		*H*	−	−	−	+	−	+	NA	−	−	−
SHO	O.F1	*p-*value	0.0274	0.9675	1.49E−5	0.0068	0.9028	5.98E−04	9.75E−06	NA	5.06E−04	1.92E−07
		*H*	+	−	+	+	−	+	+	NA	+	+
	O.F2	*p-*value	2.30E−05	0.0014	4.90E−01	5.23E−07	0.0002	4.33E−04	0.5161	NA	1.19E−07	1.23E−07
		*H*	+	+	−	+	+	+	−	NA	+	+
	O.F3	*p-*value	0.0002	0.0043	7.97E−01	1.06E−07	0.0012	2.47E−04	0.7557	NA	1.20E−01	2.73E−01
		*H*	+	+	−	+	+	+	−	NA	−	−
SRS	O.F1	*p-*value	4.67E−02	2.51E−03	0.007	3.06E−02	1.63E−02	6.69E−08	2.07E−02	5.06E−04	NA	1.07E−01
		*H*	+	+	+	+		+	+	+	NA	
	O.F2	*p-*value	4.36E−06	6.53E−08	0.000141	9.46E−01	6.53E−08	6.53E−08	9.07E−04	1.19E−07	NA	9.68E−01
		*H*	+	+	+	−	+	+	+	+	NA	−
	O.F3	*p-*value	1.79E−04	3.23E−01	0.107511	7.89E−08	1.08E−01	1.38E−06	9.25E−01	1.20E−01	NA	3.30E−01
		*H*	+	−	−	+	−	+	−	−	NA	−
WSO	O.F1	*p-*value	0.4407	0.5161	0.0001	2.58E−05	0.675	1.54E−06	0.9892	0.217	9.12E−07	NA
		*H*	−	−	+	+	−	+	−	−	+	−
	O.F2	*p-*value	5.23E−07	6.80E−08	2.47E−04	9.03E−01	6.80E−08	6.80E−08	0.001782	1.23E−07	9.68E−01	NA
		*H*	+	+	+	−	+	+	+	+	−	NA
	O.F3	*p-*value	1.05E−06	0.0071	2.29E−01	6.79E−08	0.0001	1.58E−06	0.9676	2.73E−01	3.30E−01	NA
		*H*	+	+	−	+	+	+	−	−	−	NA

#### The IEEE 118 bus system configuration

Table 10 outlines the incorporation of new components necessary to expand the system to meet electrical demand. These components were selected from the best-performing runs in each model. As indicated in Table 10, Model #1 requires the addition of two circuits to supply the loads, specifically along routes 77–78 and 99–100. In contrast, Model #2 did not require the installation of new circuits due to the integration of the TCSCS planning model into the TEP model. Furthermore, Table 10 highlights the importance of installing FCLs to restrict short-circuit currents to below 28 p.u.

**Table 10:** Installed projects required for the IEEE 118-bus system.

TEP model	Added circuits	No. of added TCSCs	No. added FCLs
Model #1	77–78 (1); 99–100 (1)	–	–
Model #2	No additional circuits	3	–
Model #32	No additional circuits	8	10

## Conclusions

In this study, ten recent metaheuristic algorithms developed in the years 2022 and 2023 for solving the TEP problem were evaluated across three distinct power network systems: the Garver network and the IEEE 118-bus system, a well-established benchmark system, and the Egyptian West Delta network.

Three distinct TEP models were used to conduct this analysis. The first TEP model adhered to the standard TEP model, focusing on the optimal placement of new transmission lines and generation units. Subsequently, the model was expanded by incorporating the planning model of TCSCs in the second model, thereby increasing the number of decision-making variables. In the third model, the problem was further augmented in complexity by integrating the planning models of TCSCs and FCLs, thus encompassing a higher number of variables. A comprehensive comparative analysis of the considered algorithms was carried out through evaluation metrics, including assessment of best and worst solutions, average, and running time.

The findings derived from simulations and statistical analysis, including the Wilcoxon rank-sum test, revealed nuanced insights into the performance of the metaheuristic algorithms. Notably, KOA, DBO, and TTAO emerged as the top-performing algorithms, exhibiting superior performance in terms of both the best solutions when applied to solve the three models over the Garver network. However, KOA was superior in obtaining the best average value. It was lower than the best second algorithm by 1.4% for Model #1, 3.7% for Model #2, and 6.8 for Model #3%.

When the algorithms were applied to expand the WDN across the three models, KOA emerged as superior among other algorithms, excelling in both providing the best solution and achieving a lower average value. Its average value was 0.95% lower than the best second algorithm for Model #1, 0.59% for Model #2, and 0.39% for Model #3.

For the 118-bus system, KOA, SGA, and DBO were the best algorithms in obtaining the best solutions across all models. However, KOA was superior in terms of the best average value. KOA’s average value was lower than the best second algorithm by about 2.74%, 1.55%, and 2.6% for Model #1, Model #2, and Model #3, respectively.

The WSO exhibited the shortest computational times, being approximately 2–3 times faster than those of other algorithms. Despite its rapid convergence, the average solution value obtained is higher compared to KOA. In TEP, prioritizing the quality of solutions is paramount over the speed of the algorithm.

The results also demonstrated that integrating the planning model of TCSCs into the TEP was cost-effective. The planning cost was reduced by about 12.47% for the Garver network, 1.96% for the WDN, and 4.2% for the 118-bus system.

Future work will concentrate on assessing the considered algorithms in solving TEP with the presence of renewable energy sources and energy storage systems. Additionally, a new hybrid meta-heuristic algorithm will be developed to tackle the TEP problem. Furthermore, the future work will entail investigating and validating these algorithms across various scenarios and real-world datasets to strengthen these findings and ease their adoption in operational settings.

## Data Availability

The datasets generated during the current study are not publicly available due to their large size but are available from the corresponding author upon reasonable request.
